# Prevalence, trends and outcomes from smoking in elective surgical systems: a secondary analysis of a prospective observational cohort study across 442 hospitals from 29 countries across Europe

**DOI:** 10.1016/j.lanepe.2025.101282

**Published:** 2025-04-15

**Authors:** STARSurg Collaborative, Sivesh K. Kamarajah, Ieva Jakaityte, Setthasorn Ooi Zhi Yang, Omar Kouli, Kariem El-Boghdadly, Amanda Farley, James Glasbey, Dion Morton, Krishnarajah Nirantharakumar, Thomas Pinkney, David Thickett, Joyce Yeung, Aneel Bhangu, Dmitri Nepogodiev

**Keywords:** Smoking, Surgery, Complications, Outcomes, Prevention

## Abstract

**Background:**

Smoking remains a significant public health issue in Europe, with elective surgery offering a key opportunity for intervention. Knowledge gaps exist around its prevalence and the optimal timing for preoperative cessation to improve outcomes, despite current guidelines recommend smoking cessation up to six weeks prior to surgery. This study aims to address this gap in a large observational prospective cohort study of patients undergoing abdominal surgery across Europe.

**Methods:**

We performed a pre-planned secondary analysis of a prospective, international cohort study of patients undergoing elective abdominal surgery between January 24, and May 03, 2022. The primary measure was smoking status and secondary measures were prevalence by age, gender, and number of long-term conditions. Multilevel logistic regression was used to explain the relationship between preoperative smoking status on postoperative overall (primary outcome) and major (secondary outcome) complications. Three-level models were constructed with patients nested within hospitals and countries.

**Findings:**

16,327 patients from 442 hospitals across 29 countries were included. 3179 patients (19.5%, 95% confidence interval (CI): 18.9%–20.1%) were current smokers, ranging from 8.3% to 31.7% across the included countries. Rates of current smokers were higher in younger patients (18–40 vs 41–60 vs 61–80 vs ≥ 81 years: 26.8% [95% CI: 24.8%–28.9%] vs 25.3% [95% CI: 24.2%–26.4%] vs 15.1% [95% CI: 14.3%–16.0%] vs 5.2% [95% CI: 4.0%–6.8%]), male patients (compared to females: 22.1% [95% CI: 22.0%–23.1%] vs 17.2% [95% CI: 16.5%–18.0%]) and healthy adults (compared to one or two long-term conditions: 24.6% [95% CI: 23.2%–25.9%] vs 19.6% [95% CI: 18.6%–20.7%] vs 16.6% [95% CI: 15.8%–17.5%]). 30-day overall complications were higher across current (OR: 1.14; 95% CI: 1.03–1.27), ex-smoker <6 weeks (1.52, 1.10–2.10), ex-smoker 6 weeks–1 year (1.30, 1.01–1.68) and ex-smoker >1 year (1.13, 1.02–1.26) compared to never smokers. No difference was seen for 30-day major complications across these groups.

**Interpretation:**

The high prevalence of smoking among elective surgical patients, especially in younger, healthy patients, expose a need to strengthen preoperative cessation strategies. Although uncertainty exist around perioperative outcomes, smoking cessation in the perioperative window is a good opportunity to reach people for long-term health promotion. Embedding high-value strategies into elective surgical systems may serve as a model for broader healthcare reforms, leading to more efficient, preventive care across the entire health system.

**Funding:**

The Student Audit and Research in Surgery collaborative is supported with a Strategic Partnership with BJS Society. SKK was funded by the NIHR Doctoral Research Fellowship (NIHR303288).

**Research in context**

**Evidence before this study**

Tobacco smoking remains a leading risk factor globally, contributing to over 10% of all deaths and nearly 142 million years of life lost annually. Despite concerted public health measures, Europe retains the highest regional smoking prevalence, with 36% of men and 20% of women actively smoking. Within healthcare, elective activities in secondary care, including planned surgery, represent a critical but underleveraged setting for preventive interventions such as smoking cessation. Existing guidelines recommend smoking cessation up to six weeks before surgery to reduce risk of complications; however, this guidance is largely based on studies conducted over a decade ago. Evidence regarding the prevalence of smoking and its associated risks across different forms of elective care remains limited. We searched PubMed for peer reviewed papers published from inception to 01/12/2024 using the terms smok∗ OR tobacco AND elective care OR surgery, not limited to publications in English language. We identified five national cohort studies from the USA or UK describing association between smoking status and outcomes. However, no large-scale studies systematically evaluating smoking prevalence using elective surgery as a model of secondary care activity across diverse health systems in Europe.

**Added value of this study**

To our knowledge, this cohort study represents the largest, most comprehensive evaluation of smoking in elective care to date in Europe, with over 16,000 patients from 442 hospitals across 29 countries in Europe. By focusing on surgery as a high-impact, measurable form of elective care, this study highlights important disparities in smoking prevalence, including disproportionately high rates among younger, healthier patients undergoing planned surgery. The study further highlights that smoking status–both current and recent ex-smokers–are associated with increased odds of postoperative complications. These findings provide critical insights into the broader implications of smoking in elective care, suggesting that pathways designed for surgical settings may serve as a surrogate framework for smoking cessation interventions in other forms of planned care, such as diagnostic procedures or chronic disease management.

**Implications of all the available evidence**

The high prevalence of smoking among elective surgical patients highlights a unique opportunity for healthcare systems to implement targeted cessation strategies during planned care episodes. Although this study focuses on surgery, the findings are broadly applicable to other elective care settings in secondary healthcare. Surgery exemplifies how structured, scheduled care encounters can serve as effective touchpoints for intervention, enabling sustained health promotion efforts. Policymakers should consider embedding cessation pathways across various forms of elective care, beyond surgery, to achieve wider population health benefits. Future research should explore the translation of these findings into other elective care models, including outpatient diagnostics, routine specialist consultations, and chronic disease management programs, ensuring equitable and scalable cessation support across all elective care systems.

## Introduction

Tobacco smoking accounts for more than 175 million deaths globally over the past three decades.^1^ These include ischaemic heart diseases and cancers, which are leading causes of death. Although substantial progress has been made in reducing smoking prevalence in many countries, smoking remains a leading risk factor for preventable morbidity and mortality.^2^^,^^3^ More than one in ten global deaths and nearly 142 million years of life lost were attributable to smoking in 2021.^1^ Despite recent declines in smoking prevalence, Europe as a region remains to have the highest prevalence of tobacco smoking among adults (≥15 years old) in the world with an estimated 36% of men and 20% of women smoking tobacco in 2020.^4^ Across Europe, tobacco remains the largest preventable health hazard, responsible for 700,000 deaths each year.^5^ Smoking also has important effects on health-care costs, productivity, and health disparities.^6^^,^^7^ As a result, tobacco control is remains cross-cutting policy and public health priority globally, with enormous potential to improve population health.

Efforts to reduce prevalence of smoking requires a multi-pronged approach across different levels within health systems. At the macro-level, policy-driven interventions^8^^,^^9^ such as the WHO Framework Convention on Tobacco Control (WHO FCTC) was developed, comprising on evidence-based tobacco control policies to reduce the demand for and supply of tobacco. The include measures such as tobacco taxes, smoking bans in public spaces, and mass media campaigns to discourage smoking. Although evidence suggests the WHO FCTC has accelerated the implementation of tobacco control policies in different policy domains, considerable variation remains between countries in Europe.^5^^,^^9^^,^^10^ At the meso-level, healthcare institutions play a vital role in integrating smoking cessation programs into routine care pathways, such as embedding dedicated tobacco treatment services within hospital systems or primary care networks. However, implementation is often inconsistent, with limited resources and lack of leadership in many settings. At the micro level, individual-focused strategies such as counselling, nicotine replacement therapy, and digital health tools for smoking cessation are typically delivered by clinicians. Although these interventions can be highly effective, their impact is often limited by low uptake and inadequate integration with broader health system strategies. To achieve meaningful reductions in smoking rates, comprehensive action is needed across all levels, with increased emphasis on addressing the limitations in meso- and micro-level implementation to create synergistic effects.

At present, quantifying investment needed into implementing interventions or pathways within healthcare institutions are often either limited or focussed on patients presenting through emergency care systems. Therefore, the economic and societal return in implementing and evaluating interventions for smoking cessations for long-term population health in elective care remains an ongoing debate. This study aims to fill a critical gap by exploring the epidemiology of smoking status across diverse group of patients undergoing elective surgery across Europe. By examining disparities in age, gender and presence of long-term conditions, this study seeks to provide insights that could guide the development of more inclusive health policies and interventions, ensuring equitable access to advancements in detection and treatment.

## Methods

### Data source, study design and population

This was a pre-planned secondary analysis of the CArdiovaSCulAr outcomes after major abDominal surgEry (CASCADE) cohort study, which evaluated the incidence of postoperative cardiovascular complications after abdominal surgery. The CASCADE cohort study included consecutive adult patients (aged ≥18 years old) undergoing major abdominal surgeries across five surgical specialties (abdominal and/or pelvic visceral resection; formation or reversal of stoma; open vascular surgery, anterior abdominal wall hernia repair; or transplant surgery) and through any surgical approach. The exclusion criteria were trauma cases, planned day-case surgery or procedures without an abdominal incision. Hospitals performing elective and/or emergency major abdominal surgery in the UK, Republic of Ireland and Europe were eligible to enrol in the study. Eligible patients were identified by local collaborators at each participating hospital during five data collection periods from 24 January to 3 April 2022, with a 30-day follow-up on all patients.

This CASCADE cohort study consortium and data collection system were designed specifically for this study, building upon existing collaborative research networks in the UK, Republic of Ireland and Europe. The UK National Health Service (NHS) South East Scotland Research Ethics Service exempted the study from formal research registration (reference NR/161AB6; 9th September 2021). Since this is an observational study of routinely collected data, no participant informed consent process was required. Study approval was obtained by local investigators at their respective institutions, according to centre-specific procedures. This study is reported in accordance with the Strengthening the Reporting of Observational Studies in Epidemiology (STROBE) statement for observational studies (Appendix p 13–15).^11^ The full protocol^12^ and results of the main study^13^ are reported elsewhere, and further details in Appendix (p 16). In this secondary pre-planned analysis, we only included all patients undergoing elective abdominal surgery. Exclusion criteria were: (i) patients undergoing emergency surgery; and (ii) incomplete smoking status at the time of surgery.

### Data collection

For this cohort study, a pre-specified case report form (CRF) was developed drawing upon templates successfully used in prior multinational cohort studies conducted within our global collaborative research network.^14^, ^15^, ^16^ The CRF was developed in collaboration with a Study Management Group comprising senior clinicians and academics with expertise in surgery, perioperative care, and geriatric medicine. Several iterative rounds of expert review and refinement ensured that the CRF captured relevant patient- and operative-level data aligned with the pre-planned primary and secondary analyses. This process also ensured the content validity of the CRF, as it was informed by existing literature and clinical expertise. The CRF was designed for use by clinicians to record routinely collected clinical data on all eligible patients. Outcome data were collected during a standardised 30-day follow-up, which was conducted either in person, over the telephone, or through a review of case notes. The consistency and reliability of outcome data capture were safeguarded through the use of structured protocols for each follow-up method.

Given the multinational nature of the study, CRFs were translated into local languages as required. Translation was led by national study leads who possessed both clinical expertise and familiarity with research methodologies, ensuring that the translations were culturally and linguistically appropriate. While formal psychometric validation of the CRFs was not undertaken for each language and population, the CRFs were derived from instruments successfully used in prior large-scale cohort studies with similar research aims, thereby providing confidence in their reliability and consistency across different settings. To further ensure data integrity, the study leveraged the well-established research infrastructure of our global network. This included centralised data storage using the Research Electronic Data Capture (REDCap) web application^17^ hosted at the University of Birmingham (UK), which facilitated secure and standardised data management. Additionally, only data collection periods achieving >95% completeness were accepted for inclusion in the pooled analysis. However, no data validation was performed on ascertainment and accuracy, since previous studies we have conducted have highlighted robust data quality. Based on previous studies, data ascertainment and accuracy are as high as 96%.^18^, ^19^, ^20^, ^21^

### Study exposure and variables

Our main exposure variable of interest was the self-reported patient smoking status at the time of surgery, as current smoker, ex-smoker (stopped within six weeks, six weeks to one year, or more than one year before operation) and never smoker. The choice of these categories for smoking status cut-offs were deemed acceptable within current clinical practice guidelines in surgery and perioperative care. Secondary exposures included age at surgery, gender, number of long-term conditions and body mass index. Long-term health conditions were selected based on a previously published national Delphi consensus on the definitions and measurement of multimorbidity in research. This Delphi process included 150 clinicians and 25 public participants across the UK.^22^ The main exposure variable was the number of long-term health conditions, defined as none, one or two or more. Multimorbidity was defined as people living with two or more long-term health conditions.^22^ Since no data on mental health were collected in the study, long-term mental health conditions were not studied. A summary of all the long-term health conditions measured in the present study are reported in the Appendix (p 3–4, Table S1). Additional measures were American Society of Anaesthesiologists (ASA) fitness grade and operative factors such as urgency, indication, approach and contamination status of the operation were collected.

### Study outcomes

The primary outcome of the study was any complication after surgery, defined according to the Clavien-Dindo classification system.^23^ This system is well-validated internationally to standardise reporting of complications after surgery across five different levels. Grade I complications are those that do not require any form of medical treatment, grade II as complications requiring medical management, grade III as complications requiring intervention either under local or general anaesthesia and grade IV as complications requiring intensive care management. Grade V are deaths within 30-days from surgery. Secondary outcome measures were major complications within 30-days from surgery, defined as Clavien-Dindo grade III to V and postoperative cardiovascular complications.

### Sample size

Since this is a secondary pre-planned analysis, we performed a separate sample size calculation based on the primary outcome measure of 30-day overall complications. Based on a previous study,^24^ the reported overall complication rates for patients undergoing surgery who smoked and never smoked were 14.9% and 12.5%. Therefore, an indicative sample size calculation using these estimates suggests around 3220 patients per group at 80% power (14.9% vs 12.4%, α = 0.05) would be required to conclude a difference in 30-day overall complications rate between smoking status groups.

### Statistical analysis

Categorical variables were compared using the χ2 test. Non-parametric data summarised with medians and interquartile ranges and differences between the groups were tested using the Mann–Whitney U test. Parametric data were summarised with mean and standard deviation. Differences between groups were explored using a two-tailed Student's t-test (two comparator groups) or one-way Analysis of Variance (ANOVA, three or more comparator groups). We have compared baseline patient- and operative-level variables in two ways: (i) between patients across the smoking status; and (ii) between patients with and without complications within 30-days from surgery. Multilevel logistic regressions models were used to identify association between smoking status and postoperative outcomes within 30-days after major abdominal surgery, with population stratification by hospital and country of residence incorporated as random intercepts. All patients had the same follow-up time up to 30-days from surgery. Clinically plausible patient, and operative-related factors were selected a priori for inclusion in adjusted analyses by clinical experts within the Trial Management Group. The factors included into the models: age, gender, body mass index, ASA physical status, clinical frailty, number of comorbidities, indication, operative approach, wound contamination, and operative specialty.

Sensitivity analysis was performed to understand potential bias from missing data on the association between smoking status and postoperative outcomes such as any complications or major complications. To do so, we used multiple imputation with chained equations to address missing data, assuming the pattern of missingness was at random.^25^ We included the exposure, confounders and the outcome in the imputation models to ensure compatibility. We used logistic regression imputation for binary data, polytomous regression imputation for unordered categorical data and proportional odds model for ordered categorical data.^25^ We assessed convergence of the imputation models by examining convergence plots and examined the distributions of imputed values to ensure plausibility. We generated 10 multiply imputed datasets; all primary analyses were performed in each imputed dataset and model and validation parameters were pooled using Rubin's rules.^26^ A p-value of <0.05 was considered statistically significant. Data analysis was performed using R Foundation Statistical software (R 3.2.2) with TableOne, ggplot 2, Hmisc, mice, Matchit and survival packages (R Foundation for Statistical Computing, Vienna, Austria).

### Role of the funding source

The sponsor of the study had no role in study design, data collection, data analysis, data interpretation, or writing of the report. The corresponding author had full access to all the data in the study and had final responsibility for the decision to submit for publication.

## Results

### Baseline characteristics

**Fig. 1:**
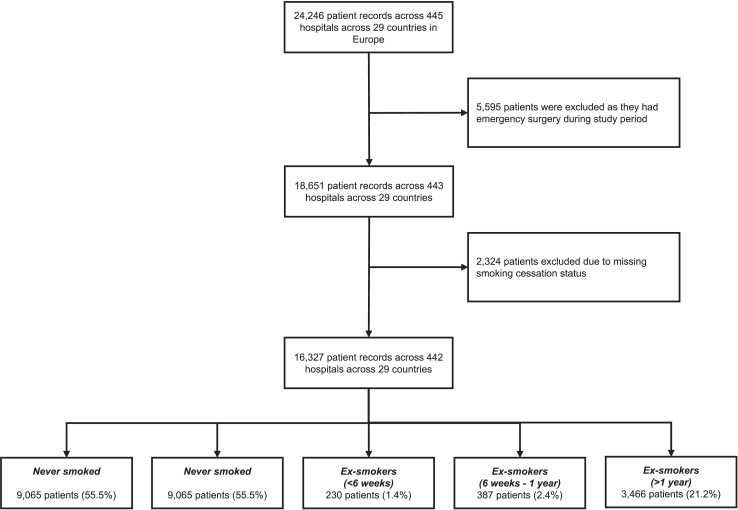
Flow chart of patients included in this secondary pre-planned analysis. Flow chart showing inclusion of 18,651 patients from 443 hospitals across 29 countries, of which 2234 patients have missing smoking status. Patients with missing smoking status were imputed in the sensitivity analysis.

Of 24,246 eligible patients undergoing major abdominal surgery, 5595 were excluded as they had emergency surgery and 2324 were excluded due to missing data on smoking status at the point of surgery. A final 16,327 patients across 442 hospitals from 29 countries were included in the final analysis. Of these patients, 3179 (19.5%) were current smokers, 230 (1.4%) were ex-smokers (<6 weeks), 387 (2.4%) were ex-smokers (up to one year) and 3466 (21.2%) were ex-smokers (more than one year). A summary flow diagram is presented in Fig. 1. Rates of current smokers vary from 8.3% to 31.7% across the 29 countries included (Appendix p 5, Table S2). Median age of the entire cohort was 62.0 (interquartile range: 50.0–71.0 years) and 4.976 (31.1%) patients were ASA grade III–V.

### Associations between smoking status and cohort characteristics

**Fig. 2:**
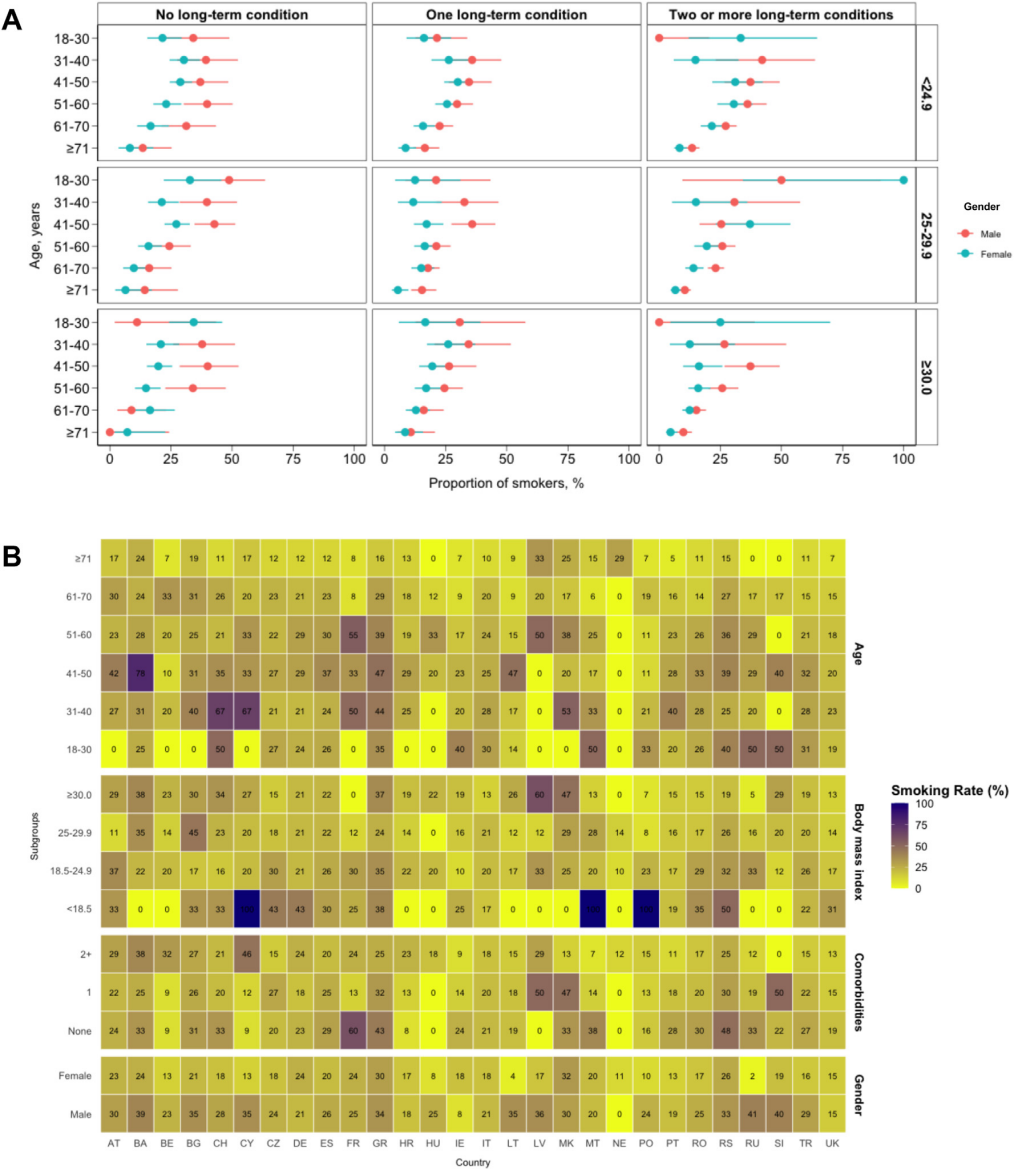
Variation in smoking rates across countries, hospitals and patient sub-groups. (A) Smoking rates across age, gender, number of long-term conditions and body mass index. (B) This heat map demonstrates the variation in rates of current smokers across different patient and operative level characteristics across the different countries included in the cohort study. Country names are provided using the ISO2 country codes. The smoking rate ranges from 0% to 100% from light yellow to dark blue.

Current smokers were more likely to be younger (18–40 years old: 15.5% vs 12.5% vs 14.3% vs 9.6% vs 4.0%) than never smokers or ex-smokers (<6 weeks vs 6 weeks to 1 year vs > 1 year), respectively (Fig. 2). They were also more likely to have ASA grade I and II (71.9% vs 71.2% vs 72.0% vs 65.4% vs 60.5%), clinical frailty score 1 to 3 (87.9% vs 86.5% vs 86.6% vs 82.2% vs 82.4%), no long-term health conditions (30.2% vs 26.1% vs 22.2% vs 17.8% vs 13.2%) compared to never smokers or ex-smokers (<6 weeks vs 6 weeks to 1 year vs > 1 year), respectively (Table 1). However, current smokers were more likely to undergo surgery for benign disease (59.4% vs 52.6%), and using open approaches (42.6% vs 39.6%) compared to never smokers (Table 2). Further, the rates of current smokers are higher in patients undergoing multi-visceral or transplant surgery, followed by gastrointestinal and liver, gynaecology and urology (22.6% vs 19.5% vs 19.1% vs 18.6%).

**Table 1:** Patient-level characteristics according to smoking status in patients undergoing elective major abdominal surgery from the CASCADE cohort study.

	Never smoked	Current	Ex-smoker (<6 weeks)	Ex-smoker (6 weeks–1 year)	Ex-smoker (>1 year)	Total	p-value
Total N (%)	9065 (55.5)	3179 (19.5)	230 (1.4)	387 (2.4)	3466 (21.2)	16,327	
Age, years							
18–40	1137 (12.5)	492 (15.5)	33 (14.3)	37 (9.6)	137 (4.0)	1836 (11.2)	<0.0001
41–60	3225 (35.6)	1481 (46.6)	80 (34.8)	139 (35.9)	937 (27.0)	5862 (35.9)	
61–80	4028 (44.4)	1153 (36.3)	109 (47.4)	201 (51.9)	2128 (61.4)	7619 (46.7)	
≥81	675 (7.4)	53 (1.7)	8 (3.5)	10 (2.6)	264 (7.6)	1010 (6.2)	
Gender							
Male	3271 (36.1)	1659 (52.2)	133 (57.8)	233 (60.2)	2209 (63.7)	7505 (46.0)	<0.0001
Female	5794 (63.9)	1520 (47.8)	97 (42.2)	154 (39.8)	1257 (36.3)	8822 (54.0)	
BMI, kg/m^2^							
<18.5	201 (2.2)	97 (3.1)	7 (3.0)	11 (2.8)	45 (1.3)	361 (2.2)	<0.0001
18.5–24.9	2995 (33.0)	1219 (38.3)	84 (36.5)	128 (33.1)	1005 (29.0)	5431 (33.3)	
25–29.9	3177 (35.0)	1070 (33.7)	86 (37.4)	142 (36.7)	1328 (38.3)	5803 (35.5)	
≥30.0	2366 (26.1)	708 (22.3)	43 (18.7)	94 (24.3)	980 (28.3)	4191 (25.7)	
(Missing)	326 (3.6)	85 (2.7)	10 (4.3)	12 (3.1)	108 (3.1)	541 (3.3)	
ASA physical status							
I	1470 (16.5)	429 (13.7)	29 (12.9)	44 (11.5)	290 (8.5)	2262 (14.1)	<0.0001
II	4863 (54.7)	1828 (58.2)	133 (59.1)	205 (53.8)	1764 (52.0)	8793 (54.8)	
III	2391 (26.9)	829 (26.4)	58 (25.8)	120 (31.5)	1249 (36.8)	4647 (29.0)	
IV/V	167 (1.9)	54 (1.7)	5 (2.2)	12 (3.1)	91 (2.7)	329 (2.1)	
Clinical frailty							
1–3	7178 (86.5)	2592 (87.9)	188 (86.6)	296 (82.2)	2602 (82.4)	12,856 (85.8)	<0.0001
4–6	1068 (12.9)	342 (11.6)	28 (12.9)	58 (16.1)	530 (16.8)	2026 (13.5)	
7–9	49 (0.6)	14 (0.5)	1 (0.5)	6 (1.7)	26 (0.8)	96 (0.6)	
Number of LTC							
0	2368 (26.1)	959 (30.2)	51 (22.2)	69 (17.8)	456 (13.2)	3903 (23.9)	<0.0001
1	2944 (32.5)	1006 (31.6)	72 (31.3)	128 (33.1)	977 (28.2)	5127 (31.4)	
≥2	3748 (41.3)	1211 (38.1)	107 (46.5)	190 (49.1)	2031 (58.6)	7287 (44.6)	
(Missing)	5 (0.1)	3 (0.1)	0 (0.0)	0 (0.0)	2 (0.1)	10 (0.1)

**Table 2:** Operative-level characteristics according to smoking status in patients undergoing major abdominal surgery from the CASCADE cohort study.

	Never smoked	Current	Ex-smoker (<6 weeks)	Ex-smoker (6 weeks–1 year)	Ex-smoker (>1 year)	Total	p-value
Total N (%)	9065 (55.5)	3179 (19.5)	230 (1.4)	387 (2.4)	3466 (21.2)	16,327	
Indication							
Benign	4764 (52.6)	1887 (59.4)	109 (47.4)	183 (47.3)	1433 (41.3)	8376 (51.3)	<0.0001
Malignant	4291 (47.3)	1290 (40.6)	120 (52.2)	204 (52.7)	2029 (58.5)	7934 (48.6)	
(Missing)	10 (0.1)	2 (0.1)	1 (0.4)	0 (0.0)	4 (0.1)	17 (0.1)	
Operative approach							
Minimally invasive	5477 (60.4)	1824 (57.4)	137 (59.6)	204 (52.7)	1944 (56.1)	9586 (58.7)	<0.0001
Open	3587 (39.6)	1355 (42.6)	93 (40.4)	183 (47.3)	1521 (43.9)	6739 (41.3)	
(Missing)	1 (0.0)	0 (0.0)	0 (0.0)	0 (0.0)	1 (0.0)	2 (0.0)	
Wound contamination							
Clean	3994 (44.1)	1460 (45.9)	98 (42.6)	163 (42.1)	1445 (41.7)	7160 (43.9)	0.03
Clean-Contaminated	4750 (52.4)	1598 (50.3)	123 (53.5)	204 (52.7)	1885 (54.4)	8560 (52.4)	
Contaminated/Dirty	301 (3.3)	119 (3.7)	9 (3.9)	19 (4.9)	130 (3.8)	578 (3.5)	
(Missing)	20 (0.2)	2 (0.1)	0 (0.0)	1 (0.3)	6 (0.2)	29 (0.2)	
Operative specialty							
GI and Liver	6161 (68.0)	2187 (68.8)	169 (73.5)	280 (72.4)	2432 (70.2)	11,229 (68.8)	<0.0001
Gynaecology	1813 (20.0)	530 (16.7)	27 (11.7)	39 (10.1)	368 (10.6)	2777 (17.0)	
Urology	759 (8.4)	292 (9.2)	22 (9.6)	41 (10.6)	456 (13.2)	1570 (9.6)	
Other (Multivisceral or Vascular)	332 (3.7)	170 (5.3)	12 (5.2)	27 (7.0)	210 (6.1)	751 (4.6)	

### Smoking status and postoperative outcomes

**Fig. 3:**
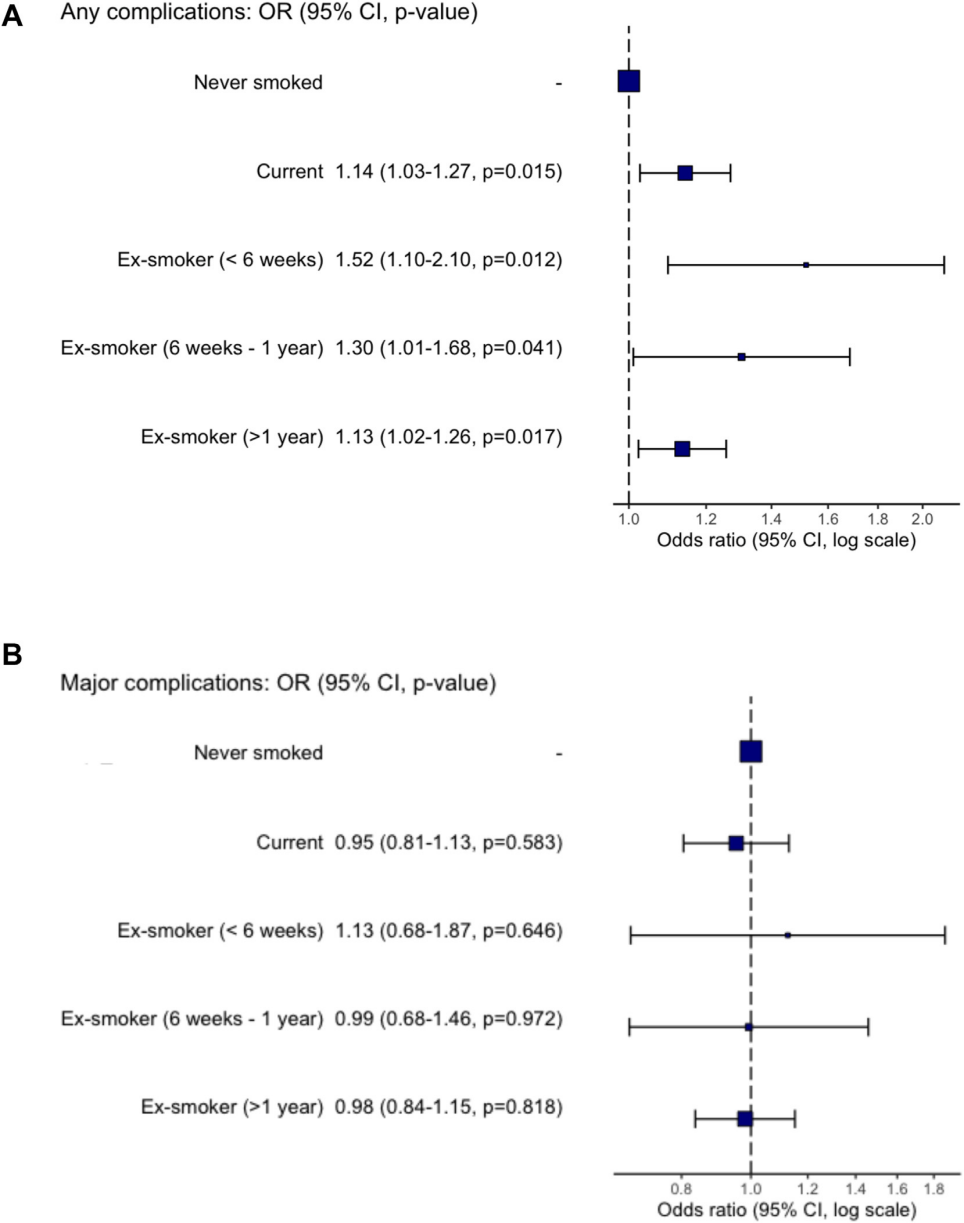
Multilevel logistic regression between smoking status and postoperative complications adjusted for confounders in patients undergoing elective abdominal surgery. (A) Any postoperative complication (B) Major postoperative complication.

A summary of the postoperative complications by smoking status in presented in Table S3 (Appendix p 6). In this cohort, 5686 patients (34.8%) experienced any complications (i.e., grade I to grade V), higher in current and ex-smokers between 6 weeks to 1-year from quitting smoking compared to never smokers (34.1% vs 42.4%) (Table S3; Appendix p 6). In a multilevel model accounting for clustering at a hospital- and country-level, the odds of any complications was significantly higher in ex-smokers (>1 year; OR: 1.13, 95% CI: 1.02–1.26), ex-smokers (six weeks to one year; OR: 1.30, 95% CI: 1.01–1.68), current smokers (OR: 1.14, 95% CI: 1.03–1.27) compared to never smokers (Fig. 3A). Full model is presented in Appendix (Table S4, p 7). Major complications occurred in 1351 patients (8.3%), higher ex-smokers (six weeks to one year) compared to current smokers and never smokers (9.3% vs 7.9% vs 7.9%) (Table S3). However, In a multilevel model accounting for clustering at a hospital- and country-level, there were no difference in major complications between ex-smokers (>1 year; OR: 0.98, 95% CI: 0.84–1.15), ex-smokers (six weeks to one year; OR: 0.99, 95% CI: 0.68–1.46), current smokers (OR: 0.95, 95% CI: 0.81–1.13) compared to never smokers (Fig. 3B). The full model is presented in the Appendix (Table S5, p 8).

### Sensitivity analysis

A sensitivity analysis was performed to understand potential bias from missing data on the association between smoking status and postoperative outcomes such as any complications or major complications (Figure S1; Appendix p 9). Missing data on smoking status was more prevalent in patients who were older (≥81 years: 7.5% vs 6.2%, p = 0.03), ASA grade I (19.0% vs 14.1%, p < 0.0001), surgery for benign disease (54.5% vs 51.3%), minimally invasive surgery (61.4% vs 58.7%, p = 0.02), clean surgery (49.1% vs 43.9%, p < 0.0001), and gynaecology procedures (19.1% vs 17.0%, p < 0.0001) (Table S6; Appendix p 10). Therefore, the missingness pattern is deemed to be missing at random. Sensitivity analysis utilised a multiple imputation approach to account for the 3529 missing data points. Adjusted models demonstrated similar findings between smoking status and postoperative any and major complications (Figures S2 & S3; Appendix p 11–12).

## Discussion

This prospective cohort study, including more than 16,000 patients from 442 hospitals across 29 countries, identifies the high prevalence of smoking rates at the time of planned elective activity using surgery as a proxy. Up to 20% of patients continue smoking, and disproportionately higher among younger, healthy adults. Substantial variation in smoking prevalence exists across countries, highlighting potential inequities in the implementation of preoperative smoking cessation strategies. Further, poor short-term outcomes amongst current smokers, exposes the urgent need to strengthen smoking cessation pathways for planned elective surgical care, where waiting times may be up to three to six months before surgery. Importantly, these interventions or pathways represent a critical opportunity for broader health promotion, addressing a modifiable risk factor to improve long-term health and reduce healthcare burdens.

At present, prevalence of current smokers in elective or planned setting can be as high as 34%.^24^^,^^27^, ^28^, ^29^, ^30^ Despite this, published studies have been limited by: (i) registries based from the USA or the UK; (ii) published more than a decade ago, thus less likely to reflect the modern surgical population; (iii) include a mix of patients presenting through elective and emergency care systems, where implementing changes in the latter are often challenging; and (iv) no clear description on the characteristics of smokers for actionable targets. Therefore, no studies have evaluated smoking prevalence in elective or planned settings in secondary care across hospitals in Europe. Only one unpublished study from the United Kingdom highlights smoking prevalence can be as high as 25% across elective and emergency settings.^31^ Although smoking prevalence was disproportionately higher among younger, healthy adults, it is possible that survivorship bias influenced our findings, as older smokers or those with significant comorbidities may have been excluded from surgery due to heightened perioperative risks. This may result in an underrepresentation of smoking prevalence in these populations. Despite this, findings of the present study do highlight the importance of targeted smoking cessation interventions in this population, who may be more receptive to behavioural modification strategies. Further, smoking rates vary across surgical specialties, consistent with previous studies showing rates as high as 25% in patients undergoing vascular surgery.^32^, ^33^, ^34^

The present study highlights that prevalence of smoking in this cohort is similar, if not higher across countries, when compared to the general population.^35^ Across Europe, the prevalence of smoking among patients undergoing elective surgery was higher than that of the general population across 16 countries. This substantial variation in smoking prevalence highlights disparities likely driven by differences in public health policies^36^^,^^37^ and socioeconomic factors.^3^ For example, countries with lower smoking prevalence often have robust tobacco control policies, such as higher taxation on tobacco products, comprehensive smoke-free laws, restrictions on tobacco advertising, and widespread availability of smoking cessation services.^36^^,^^37^ In contrast, countries with less stringent regulations or limited access to cessation support may experience higher smoking rates.^38^ Socioeconomic factors also play an important role in smoking behaviours,^39^ with smoking being more prevalent among lower-income populations.^40^ This is often driven by social stressors, targeted marketing by tobacco industries, and reduced access to educational or preventative health services. These disparities may be further amplified in low- and middle-income countries, where public health infrastructure and anti-smoking programmes are underdeveloped.^3^^,^^36^ Therefore, when comparing national smoking rates to the surgical population, it is essential to consider selection biases inherent to the surgical cohort. Certain surgeries, particularly those for conditions related to smoking (e.g., vascular or respiratory procedures), may have higher proportions of smokers. In contrast, surgeries in populations reflective of broader national demographics may align more closely with national smoking prevalence. These patterns highlight the need for targeted interventions in countries with high smoking rates, both to address the broader public health challenge and to optimise surgical outcomes through preoperative smoking cessation programmes. Addressing these disparities requires a multi-pronged approach, including strengthening tobacco control policies, integrating smoking cessation interventions into elective care pathways, and addressing the broader social determinants of health. Tailored interventions that account for country-specific smoking patterns and healthcare systems will be crucial in reducing the burden of smoking-related complications and improving equity in surgical outcomes.

Investments for strengthening smoking cessation strategies or interventions in elective care systems remain limited, particularly when compared to the substantial focus on emergency care pathways. In the UK, the long-term National Health Service plans^41^ support investment in smoking cessation pathways for inpatient settings in mental health and general medicine. This is further substantiated by high-quality research in these settings. For instance, the COSTED randomised trial^42^ conducted across six emergency departments (n = 972 patients) demonstrated a complex intervention of integrating brief advice, e-cigarette starter kits, and referrals to stop-smoking services, achieved six-month biochemical abstinence rates of 7.1% vs 4.2%. While these findings highlight the value of targeted interventions in acute settings, similar evidence in elective care is sparse. A meta-analysis^43^ of trials in elective surgery demonstrated better cessation rates lasting six to twelve months, but these studies are often small, and abstinence is rarely the primary outcome, limiting conclusions about true clinical effectiveness. However, newer, better designed research studies are shedding some light in this area.^44^, ^45^, ^46^, ^47^ For instance, a pilot randomised trial including 447 patients demonstrated providing nicotine patches when listed for surgery helped 9.1% of patients quit smoking up to four weeks before surgery.^44^ Further the use of innovative approaches such as clinical decision support system^45^ and mobile-based interventions^47^ may provide robust evidence for longer-term abstinence. However, the field is underdeveloped compared to emergency care, highlighting a critical gap in leveraging elective care settings to promote smoking cessation and improve both short- and long-term health outcomes.

As expected, patients who are smokers at the point of having surgery have poor outcomes such as 30-day overall complications, similar to the previously published studies.^24^^,^^27^, ^28^, ^29^ However, we did not observe higher odds of major complications across smokers, non-smokers and ex-smokers, despite robust adjustment. Although this study did not collect data on specific complications that may have driven the primary outcome, previous studies have shown that smokers have higher risk of having pulmonary complications or surgical site infections.^48^ However, high complication rates up to a year after quitting smoking is surprising and challenges current convention. This is because the meta-analysis from almost two decades ago have underpinned recommendations from national and international guidelines, to stop smoking up to four weeks before surgery. These observations may reflect the changing surgical population, who are increasingly older, multimorbid. Findings of this study, closely mirror those of a single-centre study from the UK including 478 patients undergoing thoracic surgery. Although underlying mechanisms are unclear, they may be related to suppressive effect of tobacco smoking on the innate immune system such as the alveolar macrophages. Tobacco has been shown to impair anti-microbial and pro-inflammatory functions of these innate cells.^49^ Further evidence suggest that recovery of these macrophages is complete at six months from smoking cessation of 6 months.^50^ These findings highlight that patients who quit smoking before surgery may need interventions such as prehabilitation to minimise their risk from surgery and not be a neglected, despite quitting more than four weeks before surgery.

The main strength of this study is the large cohort of patients across Europe involving 442 hospitals and 29 countries, By prospectively collecting data using a robust, well evaluated methodology,^14^^,^^19^ this study provides valuable insights into health systems across hospitals in the same country and different countries, identifying urgent, actionable targets for improvement. However, this study also has some important limitations to acknowledge. First, there is missing data around smoking status. To address this, we used multiple imputation as a sensitivity analysis.^51^^,^^52^ Although imperfect, this methodology is relatively robust and highlighted consistent findings. Second, we did not collect data on potential confounders such as socioeconomic status measures such as deprivation index or education attainment. Despite this, these measures are important in accounting for estimates in the multilevel models rather than the characteristics of patients undergoing surgery. Third, detailed smoking variables were not collected. These include pack-years, duration of smoking, inhalation patterns, and specific tobacco or nicotine product use (i.e., roll-your-own tobacco, electronic cigarette, heated tobacco) and reasons patient quit smoking. These factors may influence perioperative risks, and their absence limits our ability to fully evaluate their impact on clinical outcomes. Additionally, data on the precise timing of smoking cessation before surgery was not collected, as smoking status was categorised a priori during study design. As a result, sensitivity analyses exploring the continuous relationship between the timing of cessation and surgical outcomes could not be conducted. Fourth, the measurement of postoperative complications using the Clavien-Dindo classification system across multiple health systems poses challenges. Variations in how complications are managed across hospitals may influence the consistency of reporting. However, this classification system is globally validated and remains the most widely used measure of postoperative complications.^23^^,^^53^ Fifth, while formal validation of the case report form (CRF) across all 29 countries would have been ideal, we implemented measures to ensure data reliability. These included iterative CRF design, translation oversight by national leads, and rigorous data quality assurance protocols. These steps provide a strong basis for the validity of our findings despite the absence of formal validation. Finally, accounting for variation across hospitals and countries are inherently challenging in these studies of this scale. Differences in care pathways and protocols between hospitals may likely contribute to unmeasured variability. Moreover, we did not collect data on whether patients were offered any smoking cessation interventions or referred to smoking cessation services. Despite this, high rates of smokers at the point of surgery across hospitals and countries, highlight the need to strengthen systems urgently.

This study has implications for future research. First, improving implementation of existing evidence into routine elective care is important to help reduce smoking rates. At present, variation in implementation is due to the lack of clear, practical guidelines for frontline clinicians. Therefore, testing new pathways in elective care systems through research is needed to understand clinical- and cost-impacts to patients, health systems and to the population. Importantly, measuring long-term smoking cessation rates is crucial to understand success of these pathways or strategies. Second, there needs to be a clear focus on primary outcome measures for clinical trials, informed by engagement from the patients, healthcare professionals and policy makers. From a policy perspective, implementation of a structured approach to smoking cessation before surgery requires coordinated effort between surgical teams, anaesthetists, primary care physicians, and smoking cessation specialists. This preventive model may include: (i) early identification of smokers at the point of surgical referral^54^^,^^55^; (ii) routine integration of smoking cessation counselling into preoperative assessments^56^; (iii) improving access to pharmacological aids such as nicotine replacement therapy, varenicline, or bupropion (iv) leveraging digital health interventions, including smartphone apps and telehealth support^57^; and (v) ensuring continued follow-up and reinforcement of cessation efforts during postoperative recovery.^43^ Notably, in younger patients, targeted interventions such as educational campaigns, combined with behavioural support and incentives, could increase engagement with cessation services.^58^

More broadly, smokers may engage with healthcare systems at multiple touchpoints before major surgery, including visits to primary care for routine check-ups and emergency department visits. However, these encounters are often missed opportunities for smoking cessation interventions. While smoking status is frequently documented, proactive counselling and treatment for cessation are inconsistently provided. Embedding smoking cessation into routine healthcare interactions could significantly enhance preoperative risk reduction.^59^ This could be achieved by implementing systematic smoking cessation screening across all healthcare encounters,^60^ integrating automated referral pathways to cessation services,^61^^,^^62^ and training healthcare professionals, including primary care physicians, occupational health specialists, and emergency medicine clinicians to provide brief but impactful smoking cessation advice.^42^^,^^63^ A broader public health approach should also be considered, incorporating workplace health checks,^64^ digital reminders linked to electronic health records,^65^ and national-level campaigns that reinforce the role of routine healthcare encounters in promoting smoking cessation. Future research should explore strategies to integrate and evaluate these interventions within different healthcare systems globally.

The high prevalence of smoking among elective surgical patients, especially in younger, healthy patients, expose a need to strengthen preoperative cessation strategies. Although uncertainty exist around perioperative outcomes, smoking cessation in the perioperative window is a good opportunity to reach people for long-term health promotion. Embedding high-value strategies into elective surgical systems may serve as a model for broader healthcare reforms, leading to more efficient, preventive care across the entire health system.

## Contributors

The writing group and the statistical analysis group (SKK, AB, OK, DN) contributed to data curation and interpretation. The writing group contributed to writing and critical revision of the manuscript. The steering group, and national leads contributed to study conception, protocol development, study delivery, and management. The collaborators contributed to data collection and study governance across included sites. SKK, AB, OK, and DN, verified the underlying data in the study. The writing committee and the corresponding author (SK) had final responsibility for the decision to submit for publication. Detailed role descriptions of all contributing collaborating authors are shown in the Appendix.

## Data sharing statement

Data sharing requests will be considered by the writing group upon written request to the corresponding authors.

## Declaration of interests

There are no conflicts of interest to declare.

## Appendix A Supplementary data

Supplementary Tables, Figures and Collaborators group
