# Supplementary material for: Prevalence, trends and outcomes from smoking in elective surgical systems: a secondary analysis of a prospective observational cohort study across 442 hospitals from 29 countries across Europe
Source: Lancet Reg Health Eur. 2025 Apr 15;53:101282. doi: 10.1016/j.lanepe.2025.101282 (PMC12237753; doi:10.1016/j.lanepe.2025.101282)
Supplement: Supplementary Tables, Figures and Collaborators group [file mmc1.docx]

**Prevalence, trends and outcomes from smoking in elective surgical systems: A pre-planned secondary analysis of a prospective cohort study across 442 hospitals from 29 countries across Europe**

*STARSurg Collaborative*

**Collaborating authors are available in the Appendix*

**Corresponding author**: Mr Sivesh Kathir Kamarajah, NIHR Doctoral Fellow, NIHR Global Health Research Unit on Global Surgery, Department of Applied Health Sciences, School of Health Sciences, College of Medicine and Health, University of Birmingham, Birmingham, United Kingdom. Email: [s.k.kamarajah@bham.ac.uk](mailto:s.k.kamarajah@bham.ac.uk)

**Word count:**4,663

**Abstract word count:**388

**Keywords:** smoking; surgery; complications; outcomes; prevention

**Short title:** Smoking prevalence in surgery across Europe

**Conflict of interest:** There are no conflicts of interest to declare.

**Funding:** The Student Audit and Research in Surgery collaborative is supported with a Strategic Partnership with BJS Society and sponsored by the Royal College of Surgeons of Edinburgh (no grant numbers). SKK was funded by the NIHR Doctoral Research Fellowship (NIHR303288).

**Data sharing:** Data sharing requests will be considered by the writing group upon written request to the corresponding authors.

**Conflict of interest:** There are no conflicts of interest to declare.

Table of Contents

[**Table S1.** Summary of long-term health conditions in patients undergoing elective abdominal surgery across Europe 3](#_Toc191885119)

[**Table S2.** Summary of smoking rates in patients undergoing elective surgery across the included countries, ranking from highest to lowest, compared to the general population rates. 5](#_Toc191885120)

[**Table S3.** Summary of postoperative outcomes within 30-days from surgery in patients undergoing elective abdominal surgery in CASCADE 6](#_Toc191885121)

[**Table S4.** Univariable and multivariable logistic regression with patients nested within hospital and country, on any 30-day postoperative complications in patients undergoing elective major abdominal surgery 7](#_Toc191885122)

[**Table S5.** Univariable and multivariable logistic regression with patients nested within hospital and country, on 30-day major postoperative complications in patients undergoing elective major abdominal surgery 8](#_Toc191885123)

[**Figure S1.** Missing values map for the main explanatory variable and the dependent variables by the observations 9](#_Toc191885124)

[**Table S6.** Comparison between non-missing and missing baseline characteristics in the main dataset of patients undergoing major abdominal surgery 10](#_Toc191885125)

[**Figure S2**. Multilevel logistic regression between smoking status and postoperative overall complications adjusted for confounders after multiple imputation for missing data, in patients undergoing elective abdominal surgery 11](#_Toc191885126)

[**Figure S3**. Multilevel logistic regression between smoking status and postoperative major complications adjusted for confounders after multiple imputation for missing data 12](#_Toc191885127)

[**Appendix A.** STROBE Statement; checklist of items that should be included in reports of observational studies 13](#_Toc191885128)

[**Appendix B.** CASCADE cohort study 16](#_Toc191885129)

[**Appendix C.** STARSurg and EuroSurg collaborators authorship group 17](#_Toc191885130)

[**References** 43](#_Toc191885131)

# **Table S1.** Summary of long-term health conditions in patients undergoing elective abdominal surgery across Europe

| Long-term condition | Definition | Rates, n (%) |
| --- | --- | --- |
| Diabetes mellitus | A chronic metabolic disorder characterised by persistent hyperglycaemia due to insufficient insulin production, impaired insulin action, or both. These include either type I or II diabetes. | 2659 (16.3) |
| Chronic kidney disease | A progressive decline in kidney function lasting ≥3 months, defined by a glomerular filtration rate (GFR) <60 mL/min/1.73 m² or markers of kidney damage (e.g., albuminuria). Severity ranges from stage 1 (mild kidney damage) to Stage 5 (end-stage renal disease). Complications include anaemia, electrolyte imbalances, hypertension, and cardiovascular disease. | 2764 (16.9) |
| Ischaemic heart disease | A condition caused by reduced blood supply to the heart muscle, typically due to coronary artery atherosclerosis. Clinical presentation includes stable angina or acute coronary syndrome (unstable angina, myocardial infarction). | 1142 (7.0) |
| Stroke | A clinical syndrome caused by acute disruption of blood flow to the brain, leading to neurological deficits lasting >24 hours or causing death. Common types include Ischaemic (most common, due to thrombus/embolus) or haemorrhagic (rupture of a blood vessel). Risk factors are hypertension, atrial fibrillation, diabetes, and smoking. | 450 (2.8) |
| Hypertension | A chronic condition defined by persistently elevated blood pressure (≥140/90 mmHg or lower thresholds in specific guidelines). Main types are primary (essential, no clear cause) and secondary (due to an underlying condition). Common complications are stroke, ischaemic heart disease, chronic kidney disease and heart failure. | 6495 (39.8) |
| Atrial fibrillation | A supraventricular arrhythmia characterised by disorganised atrial electrical activity, resulting in an irregular and often rapid heartbeat. Risk factors are hypertension, ischaemic heart disease, heart failure, and valvular heart disease. | 828 (5.1) |
| Chronic obstructive pulmonary disease | A group of progressive lung diseases, including chronic bronchitis and emphysema, characterised by airflow limitation that is not fully reversible. | 980 (6.0) |
| Chronic liver disease | A spectrum of liver disorders marked by progressive destruction and regeneration of liver tissue, often culminating in cirrhosis. Common causes are chronic viral hepatitis (B/C), alcohol-related liver disease, non-alcoholic fatty liver disease. Complications include portal hypertension, hepatic encephalopathy, liver failure. | 348 (2.1) |
| Congestive heart failure | A clinical syndrome resulting from the heart's inability to pump blood effectively, leading to symptoms of fluid overload and reduced perfusion. Main types are heart failure with reduced ejection fraction (HFrEF) or preserved ejection fraction (HFpEF). | 353 (2.2) |
| Cancer | A group of diseases characterised by uncontrolled growth and spread of abnormal cells that can invade tissues and metastasise. These include solid tumours (e.g., breast, lung, colon) and haematological malignancies (e.g., leukaemia, lymphoma). | 7704 (47.2) |
| Inflammatory bowel disease | A group of chronic inflammatory conditions affecting the gastrointestinal tract, primarily Crohn’s disease (CD) and ulcerative colitis (UC). Common symptoms are abdominal pain, diarrhoea (often bloody in UC), and weight loss. Complications include strictures, fistulas, and increased risk of colorectal cancer. | 723 (4.4) |
| Venous thromboembolism | A condition encompassing deep vein thrombosis (DVT) and pulmonary embolism (PE), caused by blood clot formation in the venous system. Common risk factors are immobility, malignancy or inherited thrombophilia. | 515 (3.2) |

# **Table S2.** Summary of smoking rates in patients undergoing elective surgery across the included countries, ranking from highest to lowest, compared to the general population rates.

| Country | Current, n | Total, n | Proportion of current smokers, % | Reported national rates, % |
| --- | --- | --- | --- | --- |
| Overall | 3,179 | 16,327 | 19.5 | 18.4* |
| Bosnia and Herzegovina | 26 | 82 | 31.7 | - |
| Greece | 243 | 766 | 31.7 | 23.6 |
| Republic of North Macedonia | 18 | 57 | 31.6 | - |
| Latvia | 6 | 20 | 30.0 | 22.1 |
| Serbia | 52 | 180 | 28.9 | 26.2 |
| Bulgaria | 20 | 72 | 27.8 | 28.7 |
| Austria | 25 | 96 | 26.0 | 20.2 |
| Cyprus | 8 | 32 | 25.0 | 21.2 |
| France | 11 | 45 | 24.4 | 17.8 |
| Slovenia | 5 | 21 | 23.8 | 16.6 |
| Switzerland | 34 | 146 | 23.3 | - |
| Spain | 380 | 1648 | 23.1 | 19.7 |
| Germany | 144 | 657 | 21.9 | 21.9 |
| Turkey | 471 | 2165 | 21.8 | 27.3 |
| Czech Republic | 73 | 352 | 20.7 | 19.3 |
| Romania | 130 | 636 | 20.4 | 18.7 |
| Malta | 16 | 79 | 20.3 | 19.4 |
| Italy | 551 | 2857 | 19.3 | 16.5 |
| Croatia | 20 | 113 | 17.7 | 21.8 |
| Russia | 14 | 80 | 17.5 | - |
| Belgium | 9 | 53 | 17.0 | 14.6 |
| Lithuania | 20 | 118 | 16.9 | 18.4 |
| Portugal | 143 | 886 | 16.1 | 11.5 |
| Hungary | 3 | 20 | 15.0 | 19.3 |
| United Kingdom | 698 | 4715 | 14.8 | 11.2 |
| Poland | 29 | 199 | 14.6 | 18.4 |
| Ireland | 28 | 206 | 13.6 | 13.8 |
| Netherlands | 2 | 24 | 8.3 | 14.6 |

** These rates refer to the current smokers across the EU reported in the European Health interview survey, conducted across 27 EU member states in 2019. The reported national rates were extracted from the survey. The columns in green represent lower rates of current smokers in the general population than those undergoing surgery. The columns in purple represent higher rates of current smokers in the general population than those undergoing surgery.*

# **Table S3.** Summary of postoperative outcomes within 30-days from surgery in patients undergoing elective abdominal surgery in CASCADE

|  | Never smoked | Current | Ex-smoker (<6 weeks) | Ex-smoker (6 weeks - 1 year) | Ex-smoker (>1 year) | Total | p-value |
| --- | --- | --- | --- | --- | --- | --- | --- |
| Total N (%) | 9065 (55.5) | 3179 (19.5) | 230 (1.4) | 387 (2.4) | 3466 (21.2) | 16327 |  |
| Overall complications | 2930 (32.3) | 1085 (34.1) | 95 (41.3) | 164 (42.4) | 1412 (40.7) | 5686 (34.8) | <0.001 |
| Major complications | 716 (7.9) | 252 (7.9) | 21 (9.1) | 36 (9.3) | 326 (9.4) | 1351 (8.3) | 0.066 |
| 30-day mortality | 74 (0.8) | 24 (0.8) | 1 (0.4) | 8 (2.1) | 30 (0.9) | 137 (0.8) | 0.098 |
| Cardiovascular complications | 168 (1.9) | 55 (1.7) | 4 (1.7) | 17 (4.4) | 90 (2.6) | 334 (2.0) | 0.001 |
| 30-day reoperation | 396 (4.4) | 157 (4.9) | 11 (4.8) | 19 (4.9) | 191 (5.5) | 774 (4.7) | 0.106 |
| Critical care use |  |  |  |  |  |  |  |
| Yes, planned from theatre | 1882 (20.8) | 665 (20.9) | 58 (25.2) | 105 (27.1) | 941 (27.1) | 3651 (22.4) | <0.001 |
| Yes, unplanned from theatre | 91 (1.0) | 39 (1.2) | 6 (2.6) | 13 (3.4) | 47 (1.4) | 196 (1.2) |  |
| Yes, unplanned from ward | 53 (0.6) | 26 (0.8) | 1 (0.4) | 3 (0.8) | 33 (1.0) | 116 (0.7) |  |
| Total critical care stay, days | 1.0 (1.0 to 3.0) | 2.0 (1.0 to 3.0) | 2.0 (1.0 to 4.0) | 2.0 (1.0 to 3.0) | 2.0 (1.0 to 3.0) | 2.0 (1.0 to 3.0) | 0.111 |
| Total hospital stay, days | 5.0 (2.0 to 8.0) | 5.0 (2.0 to 8.0) | 5.0 (2.0 to 8.0) | 6.0 (3.0 to 10.0) | 6.0 (3.0 to 9.0) | 5.0 (2.0 to 8.0) | <0.001 |

# **Table S4.** Univariable and multivariable logistic regression with patients nested within hospital and country, on any 30-day postoperative complications in patients undergoing elective major abdominal surgery

|  |  | No | Yes | OR (univariable) | OR (multivariable full) |
| --- | --- | --- | --- | --- | --- |
| Smoking status | Never smoked | 6135 (67.7) | 2930 (32.3) | - | - |
|  | Current | 2094 (65.9) | 1085 (34.1) | 1.08 (1.00-1.18, p=0.062) | 1.14 (1.03-1.27, p=0.015) |
|  | Ex-smoker (< 6 weeks) | 135 (58.7) | 95 (41.3) | 1.47 (1.13-1.92, p=0.004) | 1.52 (1.10-2.10, p=0.012) |
|  | Ex-smoker (6 weeks - 1 year) | 223 (57.6) | 164 (42.4) | 1.54 (1.25-1.89, p<0.001) | 1.30 (1.01-1.68, p=0.041) |
|  | Ex-smoker (>1 year) | 2054 (59.3) | 1412 (40.7) | 1.44 (1.33-1.56, p<0.001) | 1.13 (1.02-1.26, p=0.017) |
| Age | 18-40 | 1361 (74.1) | 475 (25.9) | 0.39 (0.34-0.46, p<0.001) | 0.79 (0.63-0.98, p=0.030) |
|  | 41-60 | 4137 (70.6) | 1725 (29.4) | 0.47 (0.41-0.54, p<0.001) | 0.77 (0.65-0.92, p=0.004) |
|  | 61-80 | 4607 (60.5) | 3012 (39.5) | 0.74 (0.65-0.84, p<0.001) | 0.86 (0.73-1.01, p=0.063) |
|  | ≥81 | 536 (53.1) | 474 (46.9) | - | - |
| Gender | Male | 4497 (59.9) | 3008 (40.1) | - | - |
|  | Female | 6144 (69.6) | 2678 (30.4) | 0.65 (0.61-0.70, p<0.001) | 0.93 (0.85-1.01, p=0.099) |
| Body mass index (kg/m2) | <18.5 | 199 (55.1) | 162 (44.9) | - | - |
|  | 18.5-24.9 | 3503 (64.5) | 1928 (35.5) | 0.68 (0.55-0.84, p<0.001) | 0.82 (0.63-1.06, p=0.132) |
|  | 25-29.9 | 3820 (65.8) | 1983 (34.2) | 0.64 (0.51-0.79, p<0.001) | 0.77 (0.59-1.01, p=0.056) |
|  | ≥30.0 | 2766 (66.0) | 1425 (34.0) | 0.63 (0.51-0.79, p<0.001) | 0.82 (0.63-1.08, p=0.153) |
| ASA physical status | I | 1794 (79.3) | 468 (20.7) | - | - |
|  | II | 5898 (67.1) | 2895 (32.9) | 1.88 (1.68-2.10, p<0.001) | 1.23 (1.07-1.42, p=0.004) |
|  | III | 2626 (56.5) | 2021 (43.5) | 2.95 (2.62-3.32, p<0.001) | 1.51 (1.28-1.77, p<0.001) |
|  | IV-V | 135 (41.0) | 194 (59.0) | 5.51 (4.32-7.02, p<0.001) | 2.15 (1.58-2.93, p<0.001) |
| Clinical frailty | 1-3 | 8613 (67.0) | 4243 (33.0) | - | - |
|  | 4-6 | 1075 (53.1) | 951 (46.9) | 1.80 (1.63-1.97, p<0.001) | 1.30 (1.15-1.47, p<0.001) |
|  | 7-9 | 39 (40.6) | 57 (59.4) | 2.97 (1.97-4.47, p<0.001) | 2.10 (1.28-3.43, p=0.003) |
| Number of comorbidities | None | 3073 (78.7) | 830 (21.3) | - | - |
|  | 1 | 3404 (66.4) | 1723 (33.6) | 1.87 (1.70-2.06, p<0.001) | 1.17 (1.03-1.33, p=0.014) |
|  | 2+ | 4156 (57.0) | 3131 (43.0) | 2.79 (2.55-3.05, p<0.001) | 1.31 (1.14-1.50, p<0.001) |
| Indication | Benign | 6098 (72.8) | 2278 (27.2) | - | - |
|  | Malignant | 4528 (57.1) | 3406 (42.9) | 2.01 (1.89-2.15, p<0.001) | 1.64 (1.48-1.80, p<0.001) |
| Operative approach | Minimally-invasive | 6853 (71.5) | 2733 (28.5) | - | - |
|  | Open | 3786 (56.2) | 2953 (43.8) | 1.96 (1.83-2.09, p<0.001) | 1.93 (1.77-2.10, p<0.001) |
| Wound contamination | Clean | 5162 (72.1) | 1998 (27.9) | - | - |
|  | Clean-Contaminated | 5190 (60.6) | 3370 (39.4) | 1.68 (1.57-1.79, p<0.001) | 1.46 (1.33-1.60, p<0.001) |
|  | Contaminated/Dirty | 270 (46.7) | 308 (53.3) | 2.95 (2.48-3.50, p<0.001) | 2.40 (1.93-2.98, p<0.001) |
| Operative specialty | GI and Liver | 7050 (62.8) | 4179 (37.2) | - | - |
|  | Gynaecology | 2221 (80.0) | 556 (20.0) | 0.42 (0.38-0.47, p<0.001) | 0.58 (0.50-0.68, p<0.001) |
|  | Urology | 1034 (65.9) | 536 (34.1) | 0.87 (0.78-0.98, p=0.018) | 0.88 (0.75-1.04, p=0.143) |
|  | Other (Multivisceral or Vascular) | 336 (44.7) | 415 (55.3) | 2.08 (1.80-2.42, p<0.001) | 1.55 (1.28-1.89, p<0.001) |

**This table shows the findings of multilevel models exploring association between the exposure (i.e., smoking status) and 30-day postoperative outcomes (i.e., Clavien-Dindo overall complications). In this model, country and hospital were incorporated as random effects and the following variables identified as confounders as fixed effects: age, gender, body mass index, ASA physical status, clinical frailty, number of comorbidities, indication, operative approach, wound contamination, and operative specialty.*

**Table S5.** Univariable and multivariable logistic regression with patients nested within hospital and country, on 30-day major postoperative complications in patients undergoing elective major abdominal surgery

|  |  | No | Yes | OR (univariable) | OR (multivariable full) |
| --- | --- | --- | --- | --- | --- |
| Smoking status | Never smoked | 8343 (92.1) | 716 (7.9) | - | - |
|  | Current | 2924 (92.1) | 252 (7.9) | 1.00 (0.86-1.17, p=0.956) | 0.95 (0.81-1.13, p=0.583) |
|  | Ex-smoker (< 6 weeks) | 209 (90.9) | 21 (9.1) | 1.17 (0.74-1.85, p=0.497) | 1.13 (0.68-1.87, p=0.646) |
|  | Ex-smoker (6 weeks - 1 year) | 351 (90.7) | 36 (9.3) | 1.20 (0.84-1.70, p=0.320) | 0.99 (0.68-1.46, p=0.972) |
|  | Ex-smoker (>1 year) | 3137 (90.6) | 326 (9.4) | 1.21 (1.06-1.39, p=0.006) | 0.98 (0.84-1.15, p=0.818) |
| Age | 18-40 | 1735 (94.6) | 100 (5.4) | 0.39 (0.29-0.51, p<0.001) | 1.02 (0.73-1.43, p=0.894) |
|  | 41-60 | 5486 (93.6) | 372 (6.4) | 0.45 (0.37-0.56, p<0.001) | 0.84 (0.65-1.09, p=0.183) |
|  | 61-80 | 6866 (90.2) | 748 (9.8) | 0.73 (0.60-0.89, p=0.002) | 0.96 (0.76-1.20, p=0.700) |
|  | ≥81 | 877 (87.0) | 131 (13.0) | - | - |
| Gender | Male | 6701 (89.4) | 796 (10.6) | - | - |
|  | Female | 8263 (93.7) | 555 (6.3) | 0.57 (0.50-0.63, p<0.001) | 0.75 (0.65-0.86, p<0.001) |
| Body mass index (kg/m2) | <18.5 | 311 (86.1) | 50 (13.9) | - | - |
|  | 18.5-24.9 | 4928 (90.8) | 499 (9.2) | 0.63 (0.46-0.86, p=0.004) | 0.78 (0.55-1.11, p=0.164) |
|  | 25-29.9 | 5345 (92.2) | 450 (7.8) | 0.52 (0.38-0.72, p<0.001) | 0.65 (0.45-0.93, p=0.018) |
|  | ≥30.0 | 3885 (92.7) | 306 (7.3) | 0.49 (0.36-0.68, p<0.001) | 0.67 (0.47-0.96, p=0.031) |
| ASA physical status | I | 2172 (96.1) | 88 (3.9) | - | - |
|  | II | 8181 (93.1) | 606 (6.9) | 1.83 (1.45-2.30, p<0.001) | 1.15 (0.89-1.49, p=0.287) |
|  | III | 4080 (87.9) | 563 (12.1) | 3.41 (2.70-4.29, p<0.001) | 1.43 (1.08-1.90, p=0.012) |
|  | IV-V | 257 (78.1) | 72 (21.9) | 6.91 (4.94-9.69, p<0.001) | 2.11 (1.39-3.18, p<0.001) |
| Clinical frailty | 1-3 | 11895 (92.6) | 952 (7.4) | - | - |
|  | 4-6 | 1767 (87.3) | 258 (12.7) | 1.82 (1.58-2.11, p<0.001) | 1.26 (1.06-1.49, p=0.009) |
|  | 7-9 | 71 (74.7) | 24 (25.3) | 4.22 (2.65-6.74, p<0.001) | 2.30 (1.33-3.97, p=0.003) |
| Number of comorbidities | None | 3752 (96.2) | 147 (3.8) | - | - |
|  | 1 | 4717 (92.0) | 408 (8.0) | 2.21 (1.82-2.68, p<0.001) | 1.27 (1.01-1.60, p=0.040) |
|  | 2+ | 6485 (89.1) | 796 (10.9) | 3.13 (2.62-3.75, p<0.001) | 1.36 (1.07-1.73, p=0.012) |
| Operative indication | Benign | 7888 (94.2) | 482 (5.8) | - | - |
|  | Malignant | 7060 (89.1) | 868 (10.9) | 2.01 (1.79-2.26, p<0.001) | 1.45 (1.25-1.68, p<0.001) |
| Operative approach | Minimally-invasive | 9037 (94.3) | 545 (5.7) | - | - |
|  | Open | 5925 (88.0) | 806 (12.0) | 2.26 (2.01-2.53, p<0.001) | 1.95 (1.70-2.22, p<0.001) |
| Wound contamination | Clean | 6724 (93.9) | 433 (6.1) | - | - |
|  | Clean-Contaminated | 7723 (90.3) | 833 (9.7) | 1.67 (1.48-1.89, p<0.001) | 1.37 (1.19-1.58, p<0.001) |
|  | Contaminated/Dirty | 491 (85.7) | 82 (14.3) | 2.59 (2.01-3.34, p<0.001) | 1.74 (1.30-2.32, p<0.001) |
| Operative specialty | GI and Liver | 10180 (90.7) | 1039 (9.3) | - | - |
|  | Gynaecology | 2681 (96.6) | 95 (3.4) | 0.35 (0.28-0.43, p<0.001) | 0.57 (0.44-0.75, p<0.001) |
|  | Urology | 1477 (94.1) | 93 (5.9) | 0.62 (0.50-0.77, p<0.001) | 0.59 (0.45-0.77, p<0.001) |
|  | Other (Multivisceral or Vascular) | 626 (83.5) | 124 (16.5) | 1.94 (1.58-2.38, p<0.001) | 1.53 (1.19-1.95, p=0.001) |

**This table shows the findings of multilevel models exploring association between the exposure (i.e., smoking status) and 30-day postoperative outcomes (i.e., Clavien-Dindo major complications). In this model, country and hospital were incorporated as random effects and the following variables identified as confounders as fixed effects: age, gender, body mass index, ASA physical status, clinical frailty, number of comorbidities, indication, operative approach, wound contamination, and operative specialty.*

# **Figure S1.** Missing values map for the main explanatory variable and the dependent variables by the observations

**
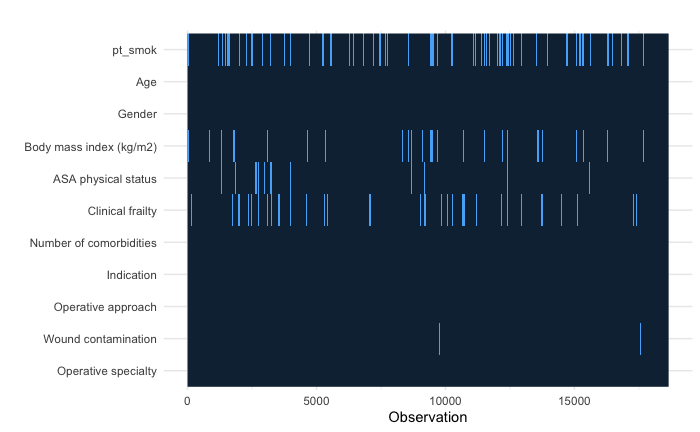
**

**Table S6.** Comparison between non-missing and missing baseline characteristics in the main dataset of patients undergoing major abdominal surgery

| Missing data analysis |  | Not missing | Missing | Total | p-value |
| --- | --- | --- | --- | --- | --- |
| Total N (%) |  | 16327 (87.5) | 2324 (12.5) | 18651 |  |
| Age | 18-40 | 1836 (11.2) | 284 (12.2) | 2120 (11.4) | 0.032 |
|  | 41-60 | 5862 (35.9) | 814 (35.0) | 6676 (35.8) |  |
|  | 61-80 | 7619 (46.7) | 1051 (45.2) | 8670 (46.5) |  |
|  | ≥81 | 1010 (6.2) | 175 (7.5) | 1185 (6.4) |  |
| Gender | Male | 7505 (46.0) | 1032 (44.4) | 8537 (45.8) | 0.164 |
|  | Female | 8822 (54.0) | 1292 (55.6) | 10114 (54.2) |  |
| Body mass index (kg/m2) | <18.5 | 361 (2.2) | 38 (1.6) | 399 (2.1) | 0.063 |
|  | 18.5-24.9 | 5431 (33.3) | 721 (31.0) | 6152 (33.0) |  |
|  | 25-29.9 | 5803 (35.5) | 667 (28.7) | 6470 (34.7) |  |
|  | ≥30.0 | 4191 (25.7) | 520 (22.4) | 4711 (25.3) |  |
|  | (Missing) | 541 (3.3) | 378 (16.3) | 919 (4.9) |  |
| ASA physical status | I | 2262 (14.1) | 429 (19.0) | 2691 (14.7) | <0.001 |
|  | II | 8793 (54.8) | 1171 (51.9) | 9964 (54.5) |  |
|  | III | 4647 (29.0) | 612 (27.1) | 5259 (28.8) |  |
|  | IV-V | 329 (2.1) | 45 (2.0) | 374 (2.0) |  |
| Clinical frailty | 1-3 | 12856 (85.8) | 1702 (84.8) | 14558 (85.7) | 0.425 |
|  | 4-6 | 2026 (13.5) | 293 (14.6) | 2319 (13.7) |  |
|  | 7-9 | 96 (0.6) | 13 (0.6) | 109 (0.6) |  |
| Number of comorbidities | None | 3903 (23.9) | 592 (25.5) | 4495 (24.1) | 0.203 |
|  | 1 | 5127 (31.4) | 728 (31.3) | 5855 (31.4) |  |
|  | 2+ | 7287 (44.6) | 1001 (43.1) | 8288 (44.4) |  |
|  | (Missing) | 10 (0.1) | 3 (0.1) | 13 (0.1) |  |
| Indication | Benign | 8376 (51.3) | 1267 (54.5) | 9643 (51.7) | 0.005 |
|  | Malignant | 7934 (48.6) | 1057 (45.5) | 8991 (48.2) |  |
|  | (Missing) | 17 (0.1) | 0 (0.0) | 17 (0.1) |  |
| Operative approach | Minimally-invasive | 9586 (58.7) | 1426 (61.4) | 11012 (59.0) | 0.016 |
|  | Open | 6739 (41.3) | 898 (38.6) | 7637 (40.9) |  |
|  | (Missing) | 2 (0.0) | 0 (0.0) | 2 (0.0) |  |
| Wound contamination | Clean | 7160 (43.9) | 1140 (49.1) | 8300 (44.5) | <0.001 |
|  | Clean-Contaminated | 8560 (52.4) | 1091 (46.9) | 9651 (51.7) |  |
|  | Contaminated/Dirty | 578 (3.5) | 91 (3.9) | 669 (3.6) |  |
|  | (Missing) | 29 (0.2) | 2 (0.1) | 31 (0.2) |  |
| Operative specialty | GI and Liver | 11229 (68.8) | 1603 (69.0) | 12832 (68.8) | <0.001 |
|  | Gynaecology | 2777 (17.0) | 445 (19.1) | 3222 (17.3) |  |
|  | Urology | 1570 (9.6) | 207 (8.9) | 1777 (9.5) |  |
|  | Other (Multivisceral or Vascular) | 751 (4.6) | 69 (3.0) | 820 (4.4) |  |
| Any complications | No | 10641 (65.2) | 1592 (68.5) | 12233 (65.6) | 0.002 |
|  | Yes | 5686 (34.8) | 732 (31.5) | 6418 (34.4) |  |
| Major complications | No | 14964 (91.7) | 2138 (92.0) | 17102 (91.7) | 0.586 |
|  | Yes | 1351 (8.3) | 184 (7.9) | 1535 (8.2) |  |
|  | (Missing) | 12 (0.1) | 2 (0.1) | 14 (0.1) |  |

**Figure S2**. Multilevel logistic regression between smoking status and postoperative overall complications adjusted for confounders after multiple imputation for missing data, in patients undergoing elective abdominal surgery
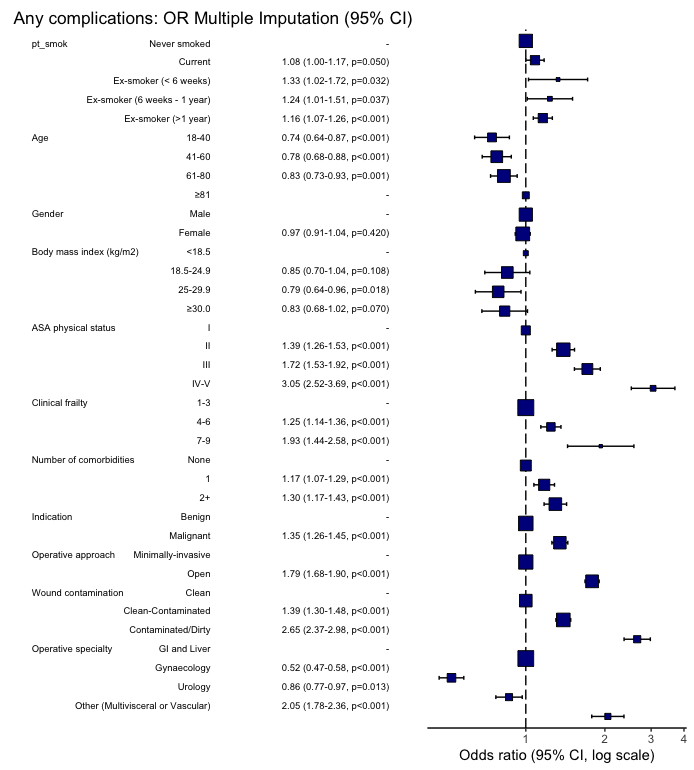


**This table shows the findings of multilevel models exploring association between the exposure (i.e., smoking status) and 30-day postoperative outcomes (i.e., Clavien-Dindo overall complications). In this model, country and hospital were incorporated as random effects and the following variables identified as confounders as fixed effects: age, gender, body mass index, ASA physical status, clinical frailty, number of comorbidities, indication, operative approach, wound contamination, and operative specialty.*

**
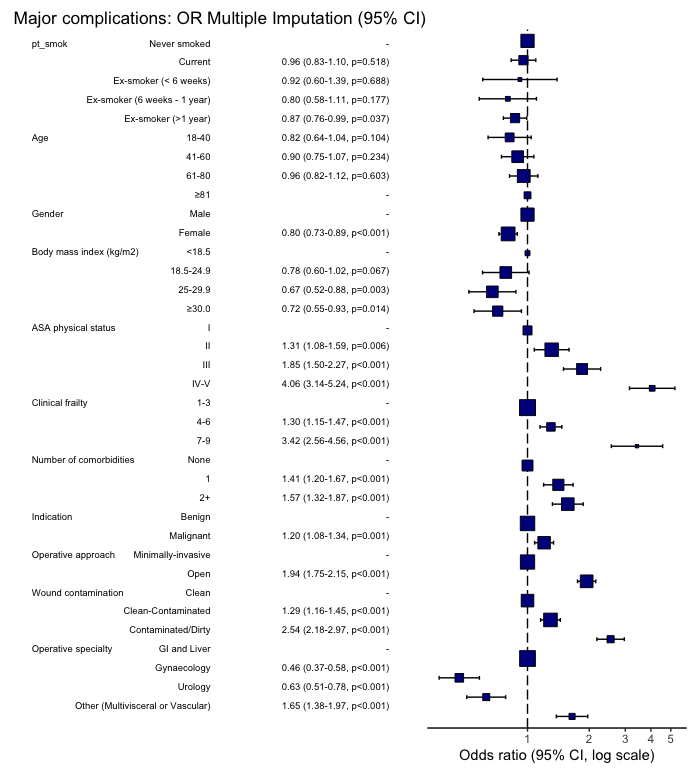
Figure S3**. Multilevel logistic regression between smoking status and postoperative major complications adjusted for confounders after multiple imputation for missing data

**This table shows the findings of multilevel models exploring association between the exposure (i.e., smoking status) and 30-day postoperative outcomes (i.e., Clavien-Dindo major complications). In this model, country and hospital were incorporated as random effects and the following variables identified as confounders as fixed effects: age, gender, body mass index, ASA physical status, clinical frailty, number of comorbidities, indication, operative approach, wound contamination, and operative specialty.*

# **Appendix A.** STROBE Statement; checklist of items that should be included in reports of observational studies

|  | Item No. | Recommendation | Page  No. |
| --- | --- | --- | --- |
| **Title and abstract** | 1 | (*a*) Indicate the study’s design with a commonly used term in the title or the abstract | 1 |
|  |  | (*b*) Provide in the abstract an informative and balanced summary of what was done and what was found | 2-3 |
| Introduction | | | |
| Background/rationale | 2 | Explain the scientific background and rationale for the investigation being reported | 6 |
| Objectives | 3 | State specific objectives, including any prespecified hypotheses | 6-7 |
| Methods | | | |
| Study design | 4 | Present key elements of study design early in the paper | 8 |
| Setting | 5 | Describe the setting, locations, and relevant dates, including periods of recruitment, exposure, follow-up, and data collection | 8 |
| Participants | 6 | (*a*) *Cohort study*—Give the eligibility criteria, and the sources and methods of selection of participants. Describe methods of follow-up  *Case-control study*—Give the eligibility criteria, and the sources and methods of case ascertainment and control selection. Give the rationale for the choice of cases and controls  *Cross-sectional study*—Give the eligibility criteria, and the sources and methods of selection of participants | 8 |
|  |  | (*b*) *Cohort study*—For matched studies, give matching criteria and number of exposed and unexposed  *Case-control study*—For matched studies, give matching criteria and the number of controls per case |  |
| Variables | 7 | Clearly define all outcomes, exposures, predictors, potential confounders, and effect modifiers. Give diagnostic criteria, if applicable | 9-10 |
| Data sources/ measurement | 8* | For each variable of interest, give sources of data and details of methods of assessment (measurement). Describe comparability of assessment methods if there is more than one group | *8* |
| Bias | 9 | Describe any efforts to address potential sources of bias | 10-11 |
| Study size | 10 | Explain how the study size was arrived at | 10 |

| Quantitative variables | 11 | Explain how quantitative variables were handled in the analyses. If applicable, describe which groupings were chosen and why |  | 10-11 |
| --- | --- | --- | --- | --- |
| Statistical methods | 12 | (*a*) Describe all statistical methods, including those used to control for confounding |  | 10-11 |
|  |  | (*b*) Describe any methods used to examine subgroups and interactions |  | 10-11 |
|  |  | (*c*) Explain how missing data were addressed |  | 10-11 |
|  |  | (*d*) *Cohort study*—If applicable, explain how loss to follow-up was addressed  *Case-control study*—If applicable, explain how matching of cases and controls was addressed  *Cross-sectional study*—If applicable, describe analytical methods taking account of sampling strategy |  | NA |
|  |  | (*e*) Describe any sensitivity analyses |  | 10-11 |
| Results | | | | |
| Participants | 13* | (a) Report numbers of individuals at each stage of study—eg numbers potentially eligible, examined for eligibility, confirmed eligible, included in the study, completing follow-up, and analysed |  | 12 |
|  |  | (b) Give reasons for non-participation at each stage |  | 12 |
|  |  | (c) Consider use of a flow diagram |  | 12 |
| Descriptive data | 14* | (a) Give characteristics of study participants (eg demographic, clinical, social) and information on exposures and potential confounders |  | 12 |
|  |  | (b) Indicate number of participants with missing data for each variable of interest |  | 12 |
|  |  | (c) *Cohort study*—Summarise follow-up time (eg, average and total amount) |  | 12 |
| Outcome data | 15* | *Cohort study*—Report numbers of outcome events or summary measures over time |  | 12-13 |
|  |  | *Case-control study—*Report numbers in each exposure category, or summary measures of exposure |  |  |
|  |  | *Cross-sectional study—*Report numbers of outcome events or summary measures |  |  |
| Main results | 16 | (*a*) Give unadjusted estimates and, if applicable, confounder-adjusted estimates and their precision (eg, 95% confidence interval). Make clear which confounders were adjusted for and why they were included |  | 12-13 |
|  |  | (*b*) Report category boundaries when continuous variables were categorized |  | 12-13 |
|  |  | (*c*) If relevant, consider translating estimates of relative risk into absolute risk for a meaningful time period |  | 12-13 |

| Other analyses | 17 | Report other analyses done—eg analyses of subgroups and interactions, and sensitivity analyses |  | 13 |
| --- | --- | --- | --- | --- |
| Discussion | | | | |
| Key results | 18 | Summarise key results with reference to study objectives |  | 14 |
| Limitations | 19 | Discuss limitations of the study, taking into account sources of potential bias or imprecision. Discuss both direction and magnitude of any potential bias |  | 16-17 |
| Interpretation | 20 | Give a cautious overall interpretation of results considering objectives, limitations, multiplicity of analyses, results from similar studies, and other relevant evidence |  | 14-16 |
| Generalisability | 21 | Discuss the generalisability (external validity) of the study results |  | 17-18 |
| Other information | |  | | |
| Funding | 22 | Give the source of funding and the role of the funders for the present study and, if applicable, for the original study on which the present article is based |  | 11 |

*Give information separately for cases and controls in case-control studies and, if applicable, for exposed and unexposed groups in cohort and cross-sectional studies.

**Note:** An Explanation and Elaboration article discusses each checklist item and gives methodological background and published examples of transparent reporting. The STROBE checklist is best used in conjunction with this article (freely available on the Web sites of PLoS Medicine at http://www.plosmedicine.org/, Annals of Internal Medicine at http://www.annals.org/, and Epidemiology at http://www.epidem.com/). Information on the STROBE Initiative is available at www.strobe-statement.org.

# **Appendix B.** CASCADE cohort study

The CASCADE cohort study included consecutive adult patients (aged ≥ 18 years) undergoing major abdominal surgeries across five surgical disciplines (abdominal and/or pelvic visceral resection; formation or reversal of stoma; open vascular surgery, anterior abdominal wall hernia repair; or transplant surgery) and through any surgical approach. Eligible patients were identified by local collaborators at each participating hospital during five data collection periods from 24 January to 3 April 2022. Hospitals performing elective and/or emergency major abdominal surgery in the UK, Republic of Ireland and Europe were eligible to enrol in the study. Recruitment of hospitals was primarily through the STARSurg^1^ and EuroSurg collaborative group network, alongside open invitation through national scientific societies, individual contacts, and social media. Researchers (doctors, medical students and clinical research staff) in eligible hospitals were asked to form teams to prospectively enrol consecutive patients in up to seven, non-overlapping, pre-defined 14-day patient-inclusion periods between 24th January 2022 and 1st May 2022. Routine, anonymised data, with no change to clinical care pathways, was collected.

Adult patients (≥18 years) who underwent major abdominal surgery (procedures as defined by StEP-COMPAC definitions^2^ were eligible. Trauma, planned day-case procedures, and procedures without an abdominal incision were not eligible for inclusion. Patients were identified at the time of operation and were followed up until their death or 30 days postoperatively, whichever came first. All data was stored in a secure database on the Research Electronic Data Capture (REDCap) web application^3^ hosted by the University of Birmingham (Birmingham, UK) and only data collection periods with >95% data completeness were accepted for pooled analysis.

# **Appendix C.** STARSurg and EuroSurg collaborators authorship group

***Writing group:*** Sivesh K Kamarajah, Omar Kouli, Ieva Jakaitytė, Setthasorn Ooi Zhi Yang, Kariem El-Boghdadly, Amanda Farley, James Glasbey, Dion Morton, Krishnarajah Nirantharakumar, Thomas Pinkney, David Thickett, Joyce Yeung, Aneel Bhangu, Dmitri Nepogodiev

***Data analysis:*** Sivesh Kamarajah, Aneel Bhangu, Omar Kouli, Dmitri Nepogodiev

***Steering Committee:*** Kamarajah SK (Chair), Blanco-Colino R, Brindl N, Brown S, Cambridge WA, Chaudhry D, Gressmann K, Gujjuri RR, Hilder A, Jaffer A, Jakaityte I, Kawka M, Kouli O, Matey E, McLean KA, Mergo A, Mills EC, Murray V, Ooi SZY, Pereira IS, Riad AM, Sgrò A, Shafi SQ, Trout I, Turňa A, Varghese C, Xu W

***External advisory group:*** Kamarajah SK (Chair), Biccard BM, Docherty A, Martin J, El-Boghdadly K, Phull M, Mouton R, Bhangu A, Glasbey JC, Harrison EM, McLean KA, Smart N, Moug S, Pinkney T, Richards T

***National leads:*** Kamarajah SK (Chair), Dajti I (Albania), de Cillia M (Austria), Van Ramshorst G (Belgium), Delibegovic S (Bosnia and Herzegovina), Mughal H (Bulgaria), Mihanovic J (Croatia), Gouvas N (Cyprus), Kocian P (Czech Republic), Levesen V (Denmark), Kaupilla J (Finland), Brindl N (Germany), Merz D (Germany), Joos M (Germany), Ioannidis A (Greece), Nnaji G (Hungary), Pata F (Italy), Pellino G (Italy), Gori A (Italy), Podda M (Italy), Riboni C (Italy), Fekaj E (Kosovo), Oliver Senica S (Latvia), Dauksa A (Lithuania), Psaila J (Malta), de Ruitjer F (Netherlands), Major P (Poland), Santos I (Portugal), Sampaio Alves M (Portugal), Simões J (Portugal), Bonci EA (Romania), Pașca A (Romania), Novikova A (Russia), Kovačević B (Serbia), Milosavljevic V (Serbia), Tadic B (Serbia), Kosir J (Slovenia), Blanco-Colino R (Spain), Pérez Ajates S (Spain), Ossola Revilla ME (Spain), Papadia A (Switzerland), Riboni C (Switzerland), Gasparri ML (Switzerland), Aktas MK (Turkey), Baki BE (Turkey), Tepe MD (Turkey), Mutlu AU (Turkey),

**Regional leads (**Kamarajah SK - Chair)

**United Kingdom**: Singal A (Aston University Medical School); Osei-Bonsu J (Barts and The London School of Medicine and Dentistry); Lacey H (Brighton and Sussex Medical School); Chan SW (Cardiff University School of Medicine); Allison M (Hull York Medical School); Duah-Asante K (Imperial College London Faculty of Medicine); Chen D (Keele University School of Medicine); Ahmed N (King’s College London GKT School of Medical Education); Ejiz A (Lancaster University Medical School); Takyi C (Newcastle University School of Medical Education); Mujeeb M (Plymouth University Peninsula Schools of Medicine and Dentistry); Ravikumar N (Queen’s University Belfast School of Medicine); Khan M (Royal College of Surgeons in Ireland School of Medicine); Hayes J (St George’s, University of London); Mckenna J (Swansea University Medical School); Wang J (The University of Edinburgh Medical School); Essa N (Trinity College Dublin School of Medicine); Xianghan H (University College Cork School of Medicine); Ko L (University College Dublin School of Medicine); Aldabbagh Y (University College London Medical School); Plascevic J (University of Aberdeen School of Medicine and Dentistry); Zia N (University of Birmingham College of Medical and Dental Sciences); Ismail R (University of Bristol Medical School); Kamel Y (University of Buckingham Medical School); Epanomeritakis I (University of Cambridge School of Clinical Medicine); Tan R (University of Dundee School of Medicine); Chiu N (University of Exeter Medical School); Naeem A (University of Glasgow School of Medicine); Kakwani M (University of Leeds School of Medicine); Mehra R (University of Leicester Medical School); Feeney K (University of Limerick School of Medicine); Yan Naing CL (University of Liverpool School of Medicine); Qureshi A (University of Manchester Medical School); Richens A (University of Nottingham School of Medicine); Li H (University of Oxford Medical Sciences Division); Ahmed R (University of Sheffield Medical School); Wilson  L (University of Southampton School of Medicine); Abraha S (University of Warwick Medical School).

*Collaborating authors (*denotes Hospital Lead):*

**Albania**: Dajti I* (University hospital Koco Gliozheni).

**Austria**: Mikalauskas S*, Kahn J, Kniepeiss D, Kušar M, Belarmino A, Frenk J, Mikalauskiene I, Angelis A, Waha JE, Al-Sharafy S, Sandano M, Schemmer P (Medical University of Graz); Reiterer C*, Horvath K, Taschner A, Riss S, Harpain F, Dawoud C, Hantáková N, Adamowitsch N, Schallmeiner S, Christian T, Xu V, Kuhrn MM, Widmann KM, Capek B, Kama B, Turgut S (Medical University of Vienna); Hauptmann L*, de Cillia M, Grünbart M, Hoi H (Saint John of God Hospital Salzburg); Binder A*, Gürtler T, Riedl P, Mayer D (Universitätsklinikum Tulln).

**Belgium**: Van Belle K*, Wautelet O, Dekkers E (Europe Hospitals); Van Daele E*, Apers T*, Van Ramshorst G, Berrevoet F, Rennie N, Dries P, Panta P, Denys A, Pype W, Bauwens T, Renard N (University Hospital of Ghent); Violon S*, Stijns J, Van De Winkel T, Van Eetvelde E (UZ Brussel).

**Bosnia and Herzegovina**: Salibasic M*, Delibegovic S, Pusina S, Hodžić E, Kruščica M, Žilić S, Bicakcic E, Rovcanin A, Kulovic E, Halilovic E, Vinčević Hodžić A (Clinical Center University of Sarajevo); Kalbić E*, Delibegovic S, Mujić A, Mešan M (General hospital Travnik); Matović E*, Delibegovic S, Kesetovic A (University Clinical Center Tuzla).

**Bulgaria**: Dardanov D*, Arabadzhieva E, Markov B, Спасова К, Biji C, Oliveira N, Miroslavov V, Korukov G, Ravendran K, Shah N, Mitkov Y, Arivanandan K, Gaikwad R, Gotru S, Saraff R, Sain N, Stanchev D, Gaydarski L, Ali U, Kandathil LJ, Krishnaveni A, Ivanov I, Ganev I, Krapeshki Т, Miza M, Mumu MSK, Bashliev I, Sajjad A, Chandel L, Yordanov D (Alexandrovska University Hospital); Hristova E*, Spassov K, Tsankov T (Fifth City Hospital); Ivanov T*, Filipov E, Georgiev D, Neykov V, Daleva E, Ilieva I, Krasimirova D, Hristov H, Арсова М, Soladoye O, Biswakarma A, Raj R, Negi A, Dey A, Adidela JK, Alionye UG, Ndukwe A (Heart and Brain Hospital); Dimov R*, Ivanov V, Sulcheva S, Mitrev S, Nikolov V (Kaspela); Karamanliev M*, Dimitrov D, Yordanov A, Vladova P, Shoshkova M, Karakadieva K, Gabarski A, Shanker A, Ehtisham H, Parambi S, Tranchev L, Vasileva A, Kareem J, Ezeabasili J, Madueke-Ediae OL, Muradia T, Saini A, Rana I, Kanagaratnam R, Thomas LS, Galasyuk M, Costa JJ, Majid N, Reddy P, Abdullahi M, Koshy Thomas S, Mehta A, Biswakarma A, Raj R, Shittu S, Theophilus J, Theophilus J, Ilyas-Uddin R, Ogunleye I, Naghavi E, Kiran A, Sundar A, Joy A, Ali F, Angelova I (UMHAT 'Georgi Stranski'); Slavchev M*, Belev N, Atanasov B, Krastev P (UMHAT Eurohospital Medical University of Plovdiv); Yotsov T*, Dimitrov D, Kamenova P, Mihaylov I, Stavrov A, Vricheva A (University Hospital Medika).

**Croatia**: Šantak G* (County General Hospital Pozega); Penezić L*, Kastelan Z, Zimak Z, Saić H, Knezevic N, Cikic B, Zekulić T, Hudolin T, Juric I, Anđelić J, Kulis T, Maric M (University Hospital Centre Zagreb); Mihanovic J*, Soric T, Surić Grčić D, Blagus L, Kovačević P, Vidić I, Miočić Juran A (Zadar General Hospital).

**Cyprus**: Ergatidou C*, Gravas S, Yiallourou A, Achilleos S, Vardas M, Kokkinos G, Panagiotou C, Kyprianou M, Kyriacou K, Papatheodorou P, Konstantinou K, Onisiforou D, Tsoutsouki F, Kasapi A, Demetriou E, Stylianou A, Xenophontos E, Georgiou P, Makrides P, Pozotou I, Constantinou M, Evangelou T, Varavina A, Charitonos S, Lampi M (Nicosia General Hospital).

**Czech Republic**: Attaalla M*, Dušek T, Ndukwe M, Kuzmane L, Moorlata E, Sillah H, Kotek J, Fazal J, Ssali E, Steingauer V, Vinklerova K, Ahmad O, Rodríguez Martínez Á, Hiebert E, Srivastava M, Ramesh Babu AS, Dobra O, Akiba A, Kalou MF, Sokolová T, Nepeřená N, Novotný P, Sedlackova J, Philips F, Al Atassi H, Jose P, Sunil Nair A, Ntashamaje L, Kaddah A, Antabi MA (Fakultní Nemocnice Hradec Králové); Kocián P*, Fiala R, Harustiak T, Vesely S, Haluza A, Štekrtová A, Král V, Zdichová K, Pastor J, Gorchakov A, Novák H, Novák V, Hornak J, Havova M, Marková M, Fořtová T, Lérias Bento D, Novysedlák R, Smetana JF, Novák T, Zdobinska T, Švarc M, Hejduková E, Havlová K, Vaishevich A, Čorňáková J, Zajíc P, Francúz P, Příman O, Havlíková Š, Zemlickova B (Motol University Hospital); Whitley A*, Gurlich R, Balaz P, Tomyak I, Kútik M, Molva O, Šturc D, Belbl M, Vinklarkova T (University Hospital Kralovske Vinohrady).

**France**: Villefranque V*, Police A*, Mikhael E, Mabilia A, Charre L, Volpin E, Braham H, Montero Macías R, Krief D, Boyer De Latour A (Hôpital Simone Veil); Angeles MA*, Martinez A, Navarro A, Ferron G, Del M, Ghiani M, Migliorelli F (Institut Claudius Regaud); Danguy Des Déserts M*, Johan S, Olivier B, Andro C (Military Hospital Clermont Tonnerre (Hôpital des Armées)).

**Germany**: Von Wedel D*, Kamphues C, Thiele M, Felber J, Neudecker J, Schineis C, Lauscher JC, Stephan R, Meyer LP, Wolff C, Bombera B (Charité University Medicine); Rolinger J*, Kirschniak A, Wilhelm P, Göller S, Van Den Hil L (Kliniken Maria Hilf); Reim D*, Kießler M, Buschhaus C, Naisar S, Zahlmann N, Walter RL, Weber M, Müller K, Schweden A, Berlet M, Jorek NA (Klinikum Rechts der Isar TUM School of Medicine); Seyfried S*, Rahbari N, Birgin E, Reissfelder C, Reeg A, Téoule P, Rasbach E (Mannheim University Medical Center (Universitätsmedizin Mannheim)); Pyrgidis N*, Hatzichristodoulou G, Sokolakis I (Martha-Maria Hospital); Strotmann JJ*, Fahlbusch T, Höhn P, Hinrichs J, Horn J, Jollet S (St. Josef); Binder J*, Förtsch T, Winterstein M, Hackner D, Girard A (Universitätsklinikum Erlangen); Wittmann M*, Vilz TO, Recker F, Velten M, Massoth G, Delis A, Brugués Villalba MI, Willis M, Soltau S, Kudaliyanage S, Eymael RL, Syring I, Neubauer R, Dahmen N, Zimmermann A, Straßberger-Nerschbach N, Puskarevic A, Wang X, Peyman L, Philippi RA, Schmidt K (University Hospital Bonn); Bork U*, Schepp F, Korn S, Radulova-Mauersberger O, Von Dem Bussche J, Von Bechtolsheim F, Praetorius C, Knobloch N, Oehme F, Weitz J, Mansfeld M, Distler M (University Hospital Carl Gustav Carus); Klingler F*, Fluegen G, Dávid S, Schneider M, Baba KA (University Hospital Duesseldorf); Ronellenfitsch U*, Kleeff J, Ukkat J, Klose J, Bayram O, Sommerer M, Rebelo A (University Hospital Halle); Gockel I*, Gentsch E, Rayes N, Thieme R, Kreuser N, Aktas B, Branzan D, Seehofer D (University Hospital Leipzig); Mallmann MR*, Mallmann C, Woldu S, Balci F, Domröse C, Diekhöner M (University Hospital of Cologne).

**Greece**: Micha G*, Stroumpoulis K, Dritsoulas L, Kalopita K, Bitzi G, Thessalonikefs P ('Elena Venizelou' General and Maternity hospital of Athens); Fradelos E*, Korkolis D, Sarafi A, Manatakis DK, Tasis N, Prountzopoulou A, Tziava E (Agios Savvas Anticancer Hospital); Pikoulas K*, Paramythiotis D, Tishukov M, Papadopoulos K, Chatziantoniou G, Tzikos G, Papamichail S, Bareka S, Sarafis A (AHEPA University General Hospital); Haidopoulos D*, Angelou K, Prodromidou A, Stamatakis E, Alexakis N, Pergialiotis V, Rodolakis A, Thomakos N (Alexandra General Hospital); Memos N*, Vlahos N*, Koutalas I, Kotsis T, Kalampokas E, Chardalias L, Kontopoulou C, Lignou C, Lykourgioti P, Bagiasta A, Petrolekas A, Savranakis O, Kozonis T, Themelidi V, Mccormac-Prekeze M, Nikolaou C, Bletsa E, Melissaridou N, Flamourakis K, Rammos P, Hadjizacharias T, Provata N, Politis D, Gkioulekas AP, Massaras D, Vakos I, Antonoglou A, Dimitra P, Kotzadimitriou A, Evangelou C, Psychogios D (Aretaieion Hospital); Konstantinidis MK*, Apostolou K*, Konstantinidis K, Patelis N, Konstantinidou S (Athens Medical Center); Kokoropoulos P*, Michalopoulos N, Kratiras Z, Drakakis P, Sidiropoulos T, Papadoliopoulou M, Vassiliu P, Arkadopoulos N, Koratzanis C, Vrachnis N, Dylja E, Chatzialis I, Sampanis D, Danias N, Fotiou A, Stavros S, Petropoulou Z, Tsaousis V, Papakonstantinou D, Lykoudis P, Nastos C, Charalampopoulos A, Papiri IE, Hatzaras I (Attikon University General Hospital); Paraskevas KI* (Central Clinic of Athens); Petrakis G*, Polenta E, Kaparounaki E (Chania General Hospital 'St George'); Sotiropoulou M*, Kapiris S, Mavrodimitraki E, Paraskeva A, Kolinioti A, Psarologos M, Stergiou D, Metaxas P, Stamatis K, Kyzeridis C, Kefalou E (Evaggelismos General Hospital); Vrakopoulou GZ*, Larentzakis A, Menenakos E, Maravgaki VR, Georgiadou C, Koutrouli M, Papageorgiou A, Skandali A (Evgenideio Hospital); Tzovaras G*, Baloyiannis I, Tzortzis V, Christodoulidis G, Perivoliotis K, Arnaoutoglou E, Ntalouka MP, Chatzis A, Daponte A, Samara A, Donoudis C, Zacharoulis D (General University Hospital of Larissa); Mulita F*, Bouchagier K, Verras G, Tchabashvili L (General University Hospital of Patras); Ioannidis O*, Koltsida A, Malliora A, Paparouni D, Bitsianis S, Loutzidou L, Anestiadou E, Χατζηανεστιάδου Χ, Simeonidis S, Tekelidis A, Zapsalis K, Skalidou S, Ouzounidis N, Ntampakis G, Kelepouri A, Kontidis F, Foutsitzis V, Kontaxi O, Barakakis G, Valaroutsou OM, Athanasiou C, Kotidis E, Pramateftakis M, Mantzoros I (George Papanikolaou General Hospital of Thessaloniki); Toutouzas K*, Frountzas M, Triantafyllou T, Triantafyllou A (Hippocratio General Hospital); Dagklis T*, Katsanos G*, Petousis S*, Athanasiadis A, Tsoulfas G, Dinas K, Tsakiridis I, Mamopoulos A, Kalogiannidis I, Rao F, Christou C, Vasileiadou S, Kapetanios G, Tsakiridis N, Kopatsaris S, Papanikolaou E, Karakasi KE, Tataridou TA, Antoniadis N, Zachomitros F, Arvanitaki A, Tsakiridis K, Anemoulis M, Neiros S, Ouranos K (Hippocratio Hospital); Tampaki EC*, Maltezos C, Maltezos K, Anastasiadou C, Chaveles A, Pachi A (KAT Athens General Hospital); Tsiantoula P*, Roditis K, Antoniou A, Bessias N, Papas T, Tzamtzidou S, Maras D, Papaioannou V (Korgialenio-Benakio Hellenic Red Cross Hospital); Koukoulis G*, Bouliaris K, Skriapas K, Kontopoulos G, Doudakmanis C, Kolla C, Efthimiou M, Kalfountzos C, Mitsakou D, Kardasi C, Zourntou S, Fountarlis LI, Bakalis A, Samarinas M, Chatzilamprou PL, Migdanis A, Karvouni K, Katsiafliaka K, Arvaniti C, Papazisi AR, Markatou V, Zervas K, Marsitopoulos K (Koutlimbaneio and Triantafylleio General Hospital of Larissa); Machairas N*, Dorovinis P, Kotsifa E, Keramida MD, Schizas D, Vailas M, Syllaios A, Mela E, Hasemaki N, Skotsimara A, Katsargyris A, Kykalos S, Tomara N, Palios I, Karniadakis I, Charalabopoulos A, Sakarellos P, Davakis S, Kydonakis N, Tsourouflis G, Stamopoulos P, Laios K, Kozadinos A (Laiko University Hospital); Katsaros I*, Kontis E, Iavazzo C, Katsiaras L, Kaouras E, Manikis P, Kokkali K, Theodorou G, Dragi A, Vorgias G (Metaxa Cancer Hospital); Tzelves L*, Skolarikos A, Manolitsis I (Sismanoglio General Hospital of Athens); Spartalis M*, Tzima I, Anastasiou A, Pantelidis P, Schismenou G, Spartalis E, Zakynthinos GE (Thoracic Diseases General Hospital Sotiria); Lasithiotakis K*, Petra G, Lampou M (University Hospital of Heraklion Crete).

**Hungary**: Toth D*, Varga Z, Pósán J, Illésy L, Váradi C (Debrecen University Clinical Center).

**Italy**: Santarelli M*, Puca L, Brignone L, Lisa Marie R, Silvia L, Silvia P, Ferguglia A, Piceni C, Improta M, Assanti F, Montanari E ('Città della Salute e della Scienza' di Torino); Ettore G*, Cannone F, Cormaci L (ARNAS Garibaldi "Nesima"); Nicastro V*, De Carlis R*, Ferrari G, Giani A, Grimaldi S, Gregorio L, Mazzola M, Ripamonti L, Lorusso L, Tartufari GA, Magistro C, Benedetti A, Gonta A, Maesano J, Bertoglio CL, Giusti I, Quagli O, Brucchi F, Bevilacqua E, De Carlis L, Lauterio A, Cerchione R, Migliorini M, Incarbone N, Centonze L, Darwish SS, Vella I, Buscemi V, Podestà A (ASST Grande Ospedale Metropolitano Niguarda); Desio M*, Berselli M, Megna S, Cocozza E, Livraghi L, Quintodei V, Marchionini V, Borroni G, Peverelli C, Corbella M (ASST Settelaghi); Ferrari F*, Odicino F, Gozzini E, Cisotto F (ASST Spedali Civili Ospedale di Brescia); Baronio G*, Del Giudice R, Sala G (ASST Valcamonica Ospedale di Esine); Iacomino A*, Vennarecci G, Ferraro D, Di Martino M, Pisaniello D, Falaschi F (Azienda Ospedaliera di rilievo nazionale Antonio Cardarelli); Petagna L*, Giuliani A, Di Lascio P, Coluzzi M, Pascale G, Gallicchio P, Frezza G, Dinatale G (Azienda Ospedaliera Regionale 'San Carlo'); Cerino G*, Bottari A*, Martellucci J, Maltinti G, Scheiterle M, Fortuna L, Staderini F, Coratti F (Azienda Ospedaliera Universitaria Careggi); Veroux M*, Giaquinta A*, Roscitano G*, Volpicelli A*, Veroux P, Palumbo M, Riccioli G, Stella L, Distefano C, Gioco R, Lomeo G, Lomeo E, Cavallo M, Virgilio C, Molino C, Giambra MM, Zerbo D, Santini R, Costa S, Incognito GG, Centonze DC (Azienda Ospedaliero); Currò G*, Ammendola M, Filippo R, Ammerata G (Azienda Ospedaliero Universitaria Mater Domini); Balestri R*, Morelli L*, Tartaglia D*, Puccini M, Asta VF, Conte V, Buccianti P, Sargenti B, Boni G, Signori S, Pezzati D, Urbani L, Casale G, Taddei G, Di Franco G, Bianchini M, Carpenito C, Furbetta N, Comandatore A, Tarasco F, Guadagni S, Mastrangelo M, Gianfaldoni C, Palmeri M, Fatucchi LM, Boato AB, Gianardi D, Stingone M, Sacco L, Giaquinto S, Lami L, Iuliano M, Annunziata EA, Chiarugi M, Strambi S, Coccolini F (Azienda Ospedaliero Universitaria Pisana); Anania G*, Chiozza M, Campagnaro A, Nevoso V (Azienda Ospedaliero Universitaria San'Anna); Carbone L*, Marano L, Micheletti G, Fontani A, Malerba S, Gambelli M, Bombino A, Grassi G (Azienda Ospedaliero Universitaria Senese); Mandato VD*, Aguzzoli L, Mastrofilippo V (Azienda Unità Sanitaria Locale); Fabbri N*, Feo CV, Ginestri M, Oppici D, Torchiaro M, Pesce A (Azienda Unità Sanitaria Locale di Ferrara); Catena F*, Vallicelli C (Bufalini Hospital); Murzi V*, Podda M, Pisanu A, Campus F, Pilia T, Saba A, Piras C, Pisano M, Locci E, Marongiu P, Gessa E (Cagliari University Hospital); D'Oria Mario*, Biloslavo A*, Lepidi S, Germani P, Nappi CJ, Grando B (Cattinara University Hospital); Lauretta A*, Pollesel S, Brollo PP (Centro di Riferimento Oncologico di Aviano (CRO)); Gallo G*, Trompetto M, Realis Luc A, Tiesi V (Clinica S. Rita); Porcu A*, Perra T, Madonia M, Tedde A, Scognamillo F, Tilocca PL, Delogu D, Mucci G, Drocchi G, Tedde M, Scanu AM, Rizzo G, Fancellu A, Feo CF, Farina G, Casu R, Cossu ML, Ginesu GC, Anania M, Dessole S, Capobianco G, Petrillo M, Dessole F, Angelone I (Cliniche San Pietro); Barberis A*, Azzinnaro A, Petrungaro A, Palli E, Mina E (E.O. Ospedali Galliera); Pipitone Federico NS*, Muratore A, Calabrò M, De Zuanni M, Herranz Van Nood E (Edoardo Agnelli); Licari L*, Callari C, Di Miceli D, Viola S (FBF Buccheri La Ferla Palermo); Manigrasso M*, Caricato C*, Milone M, Anoldo P, Vertaldi S, D'amore A, De Palma GD, Marello A, Cantore CG, Chini A, Maione F, Luglio G, Pagano G, Tropeano FP, Cricrì M, Aprea G, Palomba G, Capuano M, Basile R, Bracale U, Peltrini R, Visconti M, Magno G, Di Santo Albini AG (Federico II University of Naples); Fransvea P*, Sganga G, Tropeano G, Puccioni C, Altieri G, Fico V, Mirco P, Bianchi V, Cozza V, Pepe G, Alfieri S, Rosa F, Schena CA, Laterza V, Pascale MM, Agnes S, Bianco G, Frongillo F, Ferracci F, Santullo F (Fondazione Policlinico Universitario Agostino Gemelli); Balla A*, Lepiane P, Saraceno F (Hospital "San Paolo"); Mastroianni R*, Simone G, Tuderti G (IRCCS 'Regina Elena' National Cancer Institute); Marino F*, Lantone G, Lantone G, Isernia R, Pezzolla F, Aquilino F (IRCCS 'Saverio de Bellis'); Raimondo D*, Gori A*, Seracchioli R, Rottoli M, Belvedere A, Maurino L, Russo IS, Orsini B, Maletta M, Romano A, Bernante P, Violante T, Morezzi D, Degli Esposti E, Raffone A, Poggioli G, Canavese A, Dajti G, Cardelli S, Casadio P, Arena A, Sanna G, Cuicchi D, Angelicchio LF, Catalioto C, Torre B, De Iaco P, Tesei M, De Crescenzo E, Larotonda C, Greco F, Minghetti M, Sissa L, Perrone AM, Dondi G, Di Stanislao M, Gaetani L, Serafini L, Prosperi E, Perone D, Isopi C, Bruno F, Alexa ID (IRCCS Azienda Ospedaliero); Milito P*, Lovece A, Chiappini G, Froiio C, Panici Tonucci T, Saletta G, Scardino A (IRCCS Policlinico San Donato); Belli A*, Izzo F, Patrone R, Cutolo C, Palaia R, Granata V, Albino V, Leongito M, Piccirillo M, Pasta G, Delrio P, Rega D, Rho M, Aversano A, Angelone IA, D'amico M, Di Marzo M, De Franciscis S (Istituto Nazionale Tumori IRCSS Fondazione G. Pascale); Cianci P*, Restini E, Conversano I, Scialandrone G (Lorenzo Bonomo); Di Saverio S*, Cardinali L, Travaglini G, Sebastiani E, Marziali I (Madonna del Soccorso Hospital); Bellocchia AB* (Maria Vittoria Hospital); Raimondo I*, Di Giorgio D*, Garganese G, Tondolo V, Dore A, Pacini G, Turco LC, Campennì P, Amara MA, Rubattu A, Verri D, Sole G, Bove S, Inzaina F, Franceschi F, Grande G, Pili DP, Campus F, Delogu G, Sula V, Ez Zinabi O, Congiu M, Pira E (Mater Olbia Hospital); Ercolani G*, D'acapito F, Annunziata D, Solaini L (Morgagni); Giordano A*, Cantafio S (Nuovo Ospedale di Prato); Novello S*, Romano M, Armellin C, Zanus G, Milardi F, Grossi U, Baldan R, Brizzolari M, Scolari F, Brun-Peressut A (Ospedale Ca' Foncello); Broglia A*, Martorana M, Fazzini N (Ospedale Civile di Voghera); Marafante C*, Garino M, Moggia E, Agosti SC, Battaglia A, Borello A, Birolo SL, Barone R, Mosca C, Shakhova K, Apostu AL (Ospedale degli Infermi di Rivoli); Giaccari S*, Pavanello M, Migliori E, Sambucci D (Ospedale di Conegliano); Corbellini C*, Sampietro GM, Germiniasi G, Bischeri M (Ospedale Di Rho); Colombo F*, Danelli P, Cammarata F, Zingale RS, Pezzoli I, Carbonaro M, Albanesi F (Ospedale Luigi Sacco Milano); Cozzani F*, Rossini M, De Gennaro F, Giuffrida M, Del Rio P (Ospedale Maggiore di Parma); Inama M*, Moretto G, Iudici L, Vitali A, Creciun M, Impellizzeri H, Piazza M (Ospedale Pederzoli); Biancafarina A*, De Prizio M, Malatesti R, Sulce R, Pellicano’ GA, D'ignazio A, Mariottini V, Mura G, Angelini M, Scricciolo M, Barbara F, Tofani F, Nenci L, Borgogni V, Vece C (Ospedale San Donato USL Toscana Sud Est); Sagnotta A*, Solinas L, Rossi M, Mancini S (Ospedale San Filippo Neri); Fruscio R*, Ciulli C*, Braga M, Romano F, Garancini M, Carissimi F, Vico E, Delle Grazie CDG, Negri S, Corti J, Tamini N, Cigagna L, Giordano R, Davolio A, Trezzi G, Grassi T, Procaccianti C, Nespoli LC, Pitoni L, Fogliati A, Cordaro G, Passoni P, Fantauzzi M, Villa S, Scotti MA, Di Lucca GM, Benedetti F, Masseria P, Frigerio M, Barba M, Re I, Ceresoli M, Ferraina F, Adjei Antwi SK, Fumagalli C, Di Martino G, Di Meo ML, Bazzano L (Ospedale San Gerardo); Nelli T*, Masciello F*, Canonico G, Chisci E, Adinolfi E, Borreca R, Damigella A, Di Martino C, Mottola GIL, Fontani G, Michelagnoli S (Ospedale San Giovanni di Dio); Fedi M*, Leo F, De Vincenti R, Cecchi C, Piombetti L, Giannessi S, Benedetta B, Pagani M (Ospedale San Jacopo); Epis L*, Percivale A, Malerba M (Ospedale Santa Corona); Tonini V*, Cervellera M, Shahu J, Sartarelli L (Ospedale Santissima Annunziata); Luzzi A*, Romairone E, Carrabetta S, Tuminello F, Floris F, Ré F, Marzorati S, Ballari F, Meola C, Filippelli A, Ribeca UG, Viacava A (Ospedale Villa Scassi); Lizzi V*, Montagna M, Giuliani A, Tartaglia N, Vovola F, Maffei F, Cotoia A, Pacilli M, Pavone G (Ospedali Riuniti Azienda Ospedaliera Universitaria); Berardi G*, Depalma N, Maruccio M, Spampinato MG, D'ugo S, Marchese T, Basurto F, De Giorgi C, Garritano S, Manoochehri F, Perrone F, Botrugno I, Rizzi A, Sergi W (P.O.'Vito Fazzi'); Russo GI*, Cappellani A, Cimino S, Asmundo MG, Matarazzo MG, Di Vita M (Policlinic Hospital 'G. Rodolico'); Venturelli P*, Cocorullo G, Zarcone F, Dominici DM, Salamone G, Tutino R, Corigliano A, Guercio R, Giuseppe C, D'arpa F, Melfa G, Proclamà MP, Scerrino G, Canfora I, Marcianò M, Guercio G, Barbera S, Amato C, Agostara AV, Pantuso G, Orlando G, Finocchiaro N (Policlinico 'P. Giaccone'); Picciariello A*, Altomare DF, Vincenti L, Tomasicchio G, Paradiso N, Dezi A (Policlinico di Bari); Pinotti E*, Montuori M (Policlinico San Pietro); Siragusa L*, Pathirannehalage Don C*, Sica G, Costanzo G, Usai V, Carrubba I, Pellicciaro M, Franceschilli M, Pirozzi BM, Santori F, Busso MB, Mariani Ivanikhin A, Guida AM, Sensi B, Divizia A, Blasi F, Giudice V, Orecchia L, Angelico R, Bacchiocchi G, Tariciotti L, Billeci F, Quaranta C, Cascone C, Materazzo M, Toti L, Manzia TM, Di Lorenzo N, Monaco A, Pedini D, Bagaglini G, Keçi L (Policlinico Tor Vergata Hospital); Lapolla P*, Mingoli A, Brachini G, Cirillo B, Sapienza P, Cicerchia PM, Leonardo C, Bologna E, Licari LC, Zambon M, Mazzarella G, Falasca A, Duranti G, Flammia RS, Asero V, Bernardotto A, Scarno F, Meneghini S, Giovampietro S, Binda B, Tufano A, Palombi V, De Meis E, Rocchetti M, De Padua C, Mansi M, Bruzzaniti P, Familiari P, Nottola SA (Policlinico Umberto I); Fleres F*, Cucinotta E, Biondo SA, Viscosi F, Catarsini N, Mazzeo C, Iannello M (Policlinico Universitario G. Martino of Messina); Testa V*, Gattolin A, Rimonda R, Riente F, Travaglio E, Allisiardi F, Guffanti P (Regina Montis Regalis Hospital); Ferrara F* (San Carlo Borromeo); Barbieri MR*, Puzziello A, Mollo A, Donnarumma E, Steccanella F, Oliviero A, Lopez G, Di Maio CM (San Giovanni di Dio e Ruggi d'Aragona); Olmi S*, Di Capua F, Camillo' A, Carresi A, Uccelli M, Rubicondo C (San Marco Hospital GSD); Rosati R*, Elmore U, Puccetti F, Socci D (San Raffaele Scientific Institute); Clementi M*, Giuliani A, Tontoli S, Nisi A, Grasso A, Derraj S, Di Biasi J, Tucceri Cimini I (San Salvatore Hospital); De Nunzio C*, D'annunzio S, Nacchia A, Gallo G (Sant'Andrea Hospital); Lisi G*, Spoletini D, Signore S, Menditto R, Carlini M (Sant'Eugenio Hospital); Sasia D*, Dalmasso E*, Trucco A*, Olearo E*, Giuffrida MC, Morra I, Maione M, Puppo A, Marano A, Preve G, Vercellone B, Frola E, Beltrami E, Peluttiero I, Giraudo G, Migliore M, Armentano S, Alessandria E, Daniele A, Elisa P, Bonino L, Borghese M (Santa Croce e Carle Hospital); Bono D*, Canova G, Zago M, Nicotera A, Bonomo LD, Gattoni L (Santi Pietro e Paolo); Cillara N*, Deserra A, De Lisa A, Coccollone E, Cannavera A, Cabula R, D'agostino F, Deplano M, Chillura C, Robuschi ML, Borzacchelli A, Margiani C, Murru MC (Santissima Trinità); Pellino G*, Lieto E, Cardella F, Auricchio A, Erario SMC, Del Sorbo G, Lidia O, D'ambrosio M, Menegon Tasselli F, Mastroianni S, Selvaggi F (Universitá della Campania 'Luigi Vanvitelli'); Calini G*, Terrosu G, Clocchiatti L, Morinelli V, Valotto C, Traunero F, Vizzielli G, Restaino S, De Gennaro E, Baccarani U, Andriani A, Mauro J, Frigatti P, Martin E, Pregnolato S, Driul L (University Hospital of Udine); Zullo A*, Carcano G, Latham L, Inversini D, Vigezzi A, Ietto G, Marta R, Marzorati A, Colombo EM, Garbarino S, Palamara N, Tozzi M, Piffaretti G, Franchin M, Festi LF, Odeh M, Fanelli MC, Costa J, Pappalardo V, Scorza A, Cannavò M, Ballabio A (University of Insubria Ospedale di Circolo e Fondazione Macchi (Varese)); Barina A*, Franzato B, Rubin L, Brugnolo D, De Simoni O (Veneto Institute of Oncology (IOV) IRCCS).

**Latvia**: Senica SO*, Ozoliņš A, Vjaters E, Jain N, Apse RR, Truskovs A, Apse IA, Jukonis P, Jukonis J, Tumova A, Sivapalan H, Piirtola OSS, Berga AE, Tumegård H (Paula Stradiņa Klīniskā universitātes slimnīca).

**Lithuania**: Samalavicius N*, Nutautiene V, Aliosin O, Eismontas V (Klaipeda University Hospital); Dauksa A*, Venclauskas L, Jasaitis K, Lazauskaitė D, Urbonas K, Šlenfuktas V, Grikytė I, Jokubauskas M, Simanaitis V, Dauksa Z, Gudaityte R, Riauka R, Krunkaitytė U, Svagzdys S, Šikarskė A, Pakrosnyte J (Lithuanian University of Health Sciences Kaunas Clinics); Stratilatovas E*, Piscikaite A (National Cancer Institute); Žaldokaite G*, Poskus T, Olekaitė V (Vilnius University Hospital).

**Malta**: Psaila J*, Andrejevic P, Cini C, Cassar K, Mattocks S, Sammut M, Mizzi S, Abela L, Micallef A, Pace K, Schembri B, Cini Custo R, Harmsworth M, Farrugia B, Theuma F, Gatt R, Montebello G, Spiteri N, Galea J, Vassallo L, Fava L, Ebejer A, Carabott K, Iles K, Casingena L, Apap Bologna G, Debono G, Galea J, Sammut M (Mater Dei Hospital).

**Netherlands**: Huisman S*, Leclercq W (Máxima Medical Center); De Ruijter F*, Konsten J (VieCuri Medisch Centrum).

**North Macedonia**: Adramanova D*, Nikolovski A, Otljanski A (University Surgery Clinic "Sv. Naum Ohridski").

**Poland**: Kisielewski M*, Richter K, Kłos N, Stefura T, Macheta K, Alsoubie I, Tempski J, Wolińska N, Wysocki W (5th Military Clinical Hospital); Starek E*, Nowak NG*, Tarkowski R, Wallner G, Kobak J, Mączka E, Żak K, Ziółkiewicz A, Kułak K, Langa E, Łaba Ł, Krzesińska I, Bobiński M, Frankowska K, Dziurda S, Martyna J, Radulski J, Lulko O, Tomczyk J, Jasiński M (Independent Public Teaching Hospital No 1 in Lublin); Major P*, Rymarowicz J, Zielińska Z, Siuda K, Kęska M, Wites P, Błasiak P, Sierżęga A, Kuciel J, Tomczak D (Jagiellonian University Medical College); Molasy B*, Nieroda P, Buras E (St. Alexander Hospital).

**Portugal**: Gonçalves Pereira R*, Patrocínio S, Reis S, Rolo Santos C, Moniz L, Bernardo P (Centro Hospitalar Barreiro Montijo); Osório C*, Matos L, Carvalho L, Marques Antunes J, Alçada N (Centro Hospitalar de Entre o Douro e Vouga - Hospital de São Sebastião); Marçal A*, Tenreiro N, Martins D, Leal C, Ferreira C, Marques R, Freitas F, Marques C, Melo A, Silva S, Vieira B, Fernandes U (Centro Hospitalar de Trás-Os-Montes e Alto Douro); Freire B*, Fonseca S, Reis M, Macedo Cardoso De Oliveira C, Mendes J, Carvalho M, Macedo J (Centro Hospitalar do Medio Ave); Oliveira J*, Mexedo C, Sousa Silva D, Davide J, Emanuel Do Vale Gonçalves De Castro Alves E, Ginestal M, Santos MD, Santos J, Robalo C, Valente V, Fernandes Dos Santos JP, Mesquita I, Teixeira B, Marques Monteiro M, Silva MI (Centro Hospitalar do Porto); Nunes Coelho M*, Teixeira M, Silva RL, Lopes L, Ribeiro A, Lima Da Silva C, Correia De Sá T, Martins M, Costa M, Gil CG, Barros M, Santos RF, Silva A, Pedro J (Centro Hospitalar do Tamega e Sousa); Costa M*, Ventura Antunes TA, Pascoal M, Andrade R, Duque M, Prior I, Lemos M, Pinto P, Guerreiro P (Centro Hospitalar e Universitário de Coimbra - polo HUC); Castanheira Rodrigues S*, Barbosa E, Martins AL, Pereira A, Vaz S, Fareleira A, Rocha-Neves J, Dias L, Girão De Caires F, Pereira-Neves A, Oliveira AI, Araujo Teixeira JP, Ferreira J, Almeida M, Vb Machado M, Nogueiro J, Gomes F, Campos E, Jácome F, Coutinho C, Ribeiro Dias R, Vieira De Sousa JP (Centro Hospitalar e Universitário de São João); Francisco E*, Borges C, Pereira S, Pereira C, Machado N, Calaia R, Pinto J, Corvelo Pavão T, Santos Sousa LG, Cunha F, Melo Pinto D, Pereira R, Dinis I, Ferros C, Pinheiro JL, Santos AJ, Barbosa B, Gaspar D, Pinto M, Pereira D, Araújo N, Alves B (Centro Hospitalar Tondela); Barbosa H*, Silva Araújo D, Afonso M, Guimarães B, Campos Coroa M (Centro Hospitalar Vila Nova de Gaia/Espinho); Susano MJ*, Azevedo J, Pereira M (Fundação Champalimaud); Miranda P*, Garrido R, Oliveira Mourão M, Ferreira M, Duarte A, Félix P, Antunes I, Fernandes N, Gil Corrêa Figueira C, Figueiredo I, Gututui M, Oliveira S (Hospital Beatriz Angelo); Silva A*, Madeira Martins F, Cabral A, Rodrigues CS, Gama B, Silva Borges R (Hospital da Horta); Lourenço R*, Longras AC, Araújo R, Peixoto I, Santos A, Matos D, Lopes A, Claro L (Hospital da Senhora da Oliveira); Cardoso D*, Martins A, Silvestre M, Sousa G, Santos Bessa M, Cardoso V (Hospital de São Francisco Xavier); Velez C*, Machado A, Pedroso De Lima R, Matias MI, V. Matos S, Rocha Melo M, Bolota J, Rente M, Santos A, Antunes ML, Bárbara A, Rosin E, Oliveira J, Leandro S, Damasio Cotovio M, Morgado S (Hospital do Espirito Santo); Fialho G*, Andrade N, Valente Costa Pinto F, Capote H, Morais S, Buruian M, Taré F, Santos G, Rosado D, Costa C, Mogne T, Pratas N, Cordeiro B, Brito M, Salvador D (Hospital Doutor José Maria Grande); Carvalho S*, Capella V, Carracha M, Alves R, Miranda C, Rebelo F, Teles T, Ferreira M (Hospital Dr. Antonio José de Almeida); Cismasiu B*, Costa PM, Henriques S, Trindade M, Vaz J, Gomes Vieira M, André M, Macedo A, Gonçalves Nobre JG, Prosil Sampaio J, Mira AR, Preto Barreira AL, Botelho P (Hospital Garcia de Orta); Melo Pinto D*, Quintela C, Quelhas MJ, Pereira D, Cruz A, Mesquita Guimarães C, Pinto C, Maldonado F, Sales F, Marrana F, Reis Aguiar AR, Freire L, Farias L, Faria-Costa G, Castro A, Moreira Marques T, Cardoso G (Hospital Pedro Hispano); Ribeiro J*, Fragoso M, Silva C, Vasconcelos M, Picciochi M, Sampaio Soares A, Frazão J, Miranda Pera R, Gaboleiro F, Ramalho De Almeida F, Mendes A, Afonso F, Fontaínhas J (Hospital Professor Doutor Fernando da Fonseca); Reia M*, Angel M, Aguero C, Guerrero M, Dominguez J (Hospital Santa Luzia de Elvas); Borges Da Costa I*, Ramos L, Fernandes J, Assis C (Hospital Vila Franca de Xira); Fonseca F*, Carvalhal S, Caiado A, Brito Da Silva F, Miguel B, Moniz J, Pires M, Leal B, Nunes J, Matos Sousa F (Instituto Português de Oncologia de Lisboa Francisco Gentil); Vieira Caroço T*, Romano M, Ângelo M, Rodrigues Ferreira A, Prata Saraiva AR, Mano F, Rodrigues F, Branco T, Gaspar S, Neves F, Alves M (IPO Coimbra); Ferreira Pinto AP*, Peyroteo M, Baía C, Marques M, Correia AM, Silva JO (IPO Porto); Monteiro R*, Silva-Vaz P, Gomes J, Moutinho Teixeira MJ, Brito Neves T, Meruje F (Unidade Local de Saúde de Castelo Branco).

**Republic of Ireland**: Sheahan C*, Macnamara D, Linares Gómez JE, Clare S, Walmsley A, Tiwari A, Khan T, Dowling G, Hudak K, Craig E, Dhannoon A, Saeedi S, Shah J, Jordan M, Buckley T, Engler J, Reilly M, Ariaratnam J, Hinn MA, Benn K, Petropoulos S, Hansen HS, Browne S, Keelan S, Basta M, Pornsakulpaisal R, Tan G, Niyaz AN, Lakic N, Warhust T, Chang YQ, Qaoud Y (Beaumont Hospital, Dublin); Mwipatayi D*, Kearney D, O'reilly C, Gamper Y, Dhillon R, Patel V, Gallo K, Mulvaney H, Edwards P, Tiller S, Gorecki E, Jordan M, Daswani S, Jones V, Asekomhe I, Clausen N, Khan M, Feldiorean A, Pezeshki A, Khedro T, Beyer J, Byrne G, Irfan W, Poluha A, Fager C, Gilligan-Steinberg L, Yarp J, Byrne E, Lorico-Rappa M, Hassan A, Keogh T, Denning W, Horton-Schleicher K (Connolly Hospital, Dublin); Deane T*, Haren A, Md Hamsan AD, Mukerji N, Dwivedi M, Cheah SML, Kwek PX, Arshad S, Alzaki A, Cornila I, Feizal HM, Barry M, Semar A, Gorman P, Gordon A, Mcsweeny C, Layyous N, Peiris D, Pan S (St Vincent's University Hospital); Larkin J, Umar MM, Doherty C, Mitchell C, Mcelvaney H, Sabz Ali Z, Adesokan S, Murphy A, Yeo A, Hayes L, Owens C, Crawley N, Macinnis E, Elekes S, Oderoha KE, Mac Mahon I, Moran B, Matreja P (St. James' Hospital); Conlon K, Piankova P, Farran MZ, Kashyap SV, Puntambekar M, Sampat M, Heaslip Owens S, Maheshwari A, Soo A, Butt J, O'brien K, Almutairi M (Tallaght University Hospital); Mcfeetors C*, Kerin M, Davey MG, Murray W, Nasehi A, Graham A, Mathew C, Azam S, Chua Vi Long K, Stafford-Johnson S, Ejaz D, Siddiqi Z, Kon AMH (University Hospital Galway); Parmar M*, Peirce C, Shomoye D, Alabi A, Salzberg N, Gallippi V, Aziz J, Melly C, Gill H, Yang HW, Eltahir Ibrahim SE, Rugber Singh JSK (University Hospital Limerick).

**Romania**: Bacalbasa N*, Balescu I (Clinical Hospital Dr. Ion Cantacuzino); Muresan M*, Dudric V, Imihteev I, Chelaru V, Băiceanu TM, Alexandra M, Bashimov A, Voda M, Muresan D, Varadi R, Muntean M, Macovei Ș, Checiu R, Buf D (Cluj-Napoca Municipal Hospital); Grama F*, Chitul A, Bezede C, Ciofic E (Coltea Clinical Hospital); Beuca A*, Bintintan V, Muresan D, Raluca-Cristina A, Rad R, Șușman S, Suciu C, Abu Arif J, David A, Blaga M, Olesea B, Butnariu DA, Scurtu RR, Rad R, Claudia-Mihaela P, Girlovanu M, Costina D, Drasovean R, Tartiu G, Tache S, Irina N, Streang A, Trif A, Romascan I, Stănică E, Vass E, Cucoreanu C (County Emergency Hospital); Toma EA*, Calu V, Enciu O, Laceanu A, Maria-Zamfirescu R, Matache IM, Mușat I, Piriianu DC, Georgescu AA, Miron A, Tartalea M, Al-Ugeily N, Bogdan-Gabriel B, Alexiadi A, Zaharescu A (Elias Emergency Hospital); Ciubotaru C*, Negoi I, Paunescu M, Iancu I, Pop IV, Diaconescu B, Dao-Zamfirescu P, Patel NB, Rafi S, Popescu D, Girbaciu M, Rădoi A, Breuer C, Braun AC, Stoica B, Gîlia I, Gheorghe A, Popa EC, Brichius F, Bucurica A, Negoita VM, Radu M, Bianca Ștefania R, Socol CL, Baiceanu AM (Emergency Clinical Hospital Bucharest); Bubenek Turconi SI*, Valeanu L, Girel M, Gavrila MR, Pirvu M, Morosanu B, Ioniana M, Barbu A, Cornel R, Bogdan B, Bute T, Madec L, Burla E, Balan C, Alexandru E (Emergency Institute for Cardiovascular Diseases 'Prof. Dr. C.C. Iliescu'); Tomescu D*, Popescu M, Dumitru M, Marinescu A, Petrusca I (Fundeni Clinical Institute); Pasca A*, Achimas-Cadariu P, Irimie A, Petrusan A, Puscas M, Titu S, Jiboc NM, Vlad IC, Eniu DT, Ciubotariu P, Gata VA, Rancea A, Iulia C, Morariu DS, Nistor-Ciurba CC, Gale I, Lazar G, Bonci E, Ignat F, Lisencu C, Dumitraș C, Faur FI, Butuza MO, Marian MC, Paul Luchian A (Prof. Dr Ion Chiricuta Institute of Oncology); Bocse H*, Puia IC, Andra C, Pop P, Moldovan S, Ursu S, Sorin M (Regional Institute of Gastroenterology & Hepatology "Prof Dr Octavian Fodor"); Lunca S*, Dimofte M, Musina A, Velenciuc N, Roata CE, Morarasu S (Regional institute of Oncology Iasi); Velescu M*, Mureșan F, Zaha I, Rusu IM, Mircea B, Tauberg DG (University Hospital).

**Russia**: Kirov M*, Kuzkov V, Nikonov A, Tishchenko A, Kulin D, Mironov V (City Hospital #1); Litvin A*, Gunko I, Mazhega I, Bilenko R (Immanuel Kant Baltic Federal University); Khrykov G*, Манкевич Н, Karpenko M, Zagaynov E, Shilyaev A (Leningrad Regional Clinical Oncology Dispensary); Butyrskii A*, Tatidze A, Seytnebieva Z, Aliev A, Rumyantseva M (Municipal Emegency Hospital No.6); Yanishev A*, Abelevich A, Doktorov S, Zuev K, Lazareva A, Malov A (Nizhny Novgorod regional clinical hospital); Borisov D*, Борисов Е, Андрийчук И (Northern Medical Clinical Centre of them. N.A. Semashko); Galchikova Z*, Shemetova M*, Maltsagova K (P. HERTSEN MOSCOW ONCOLOGY RESEARCH INSTITUTE); Bedzhanyan A*, Temirsultanova K, Efremov D, Volkova A, Frolova Y, Minenkova A, Petrenko K, Sumbaev A, Bredikhin M, Bedzhanyan E, Mikhailova A (Petrovsky National Research Centre of Surgery); Тен В*, Кудрявцев Ю (Private Healthcare Institution "Russian Railways-Medicine""); Novikova A*, Pavlov R, Chernykh M, Boiko N, Данилин В (Saint Petersburg State University Hospital).

**Serbia**: Stanojevic G*, Brankovic B, Nestorovic M, Milutinovic N, Vukadinović A, Vukadinović A (Clinical Center Nis); Stojanovic B*, Radovanovic D, Cvetkovic A, Radosavljevic I, Sreckovic M, Milosevic B, Markovic N, Spasic M, Pavlovic M, Lazic D, Vucic R (Clinical Centre of Kragujevac); Tadic B*, Kajmaković B*, Vilendecic Z*, Knezevic D, Bumbasirevic U, Stefanovic A, Gregoric P, Perišić Z, Micic D, Ratkovic S, Vasiljević J, Bulat PR, Jovanovic V, Mitrović O, Gunjic J, Potkonjak D, Grubor N, Reljic M, Palibrk I, Milutinovic V, Zivkovic M, Škrijelj D, Grubač Ž, Kadija S, Cerovic Popovic R, Andric L, Aleksić L, Kmezić S, Pavlovic I, Djukanovic M, Milojevic B, Dzamic Z, Petrovic M, Jeremic K, Pilic I, Milosevic B, Krstic J, Šljukić V, Veselinović M, Ivanović N, Janičić A, Jovanovic A, Cvijanovic D, Ladjevic Likic I, Radojevic M, Ćupić J, Dimitrijevic I, Miladinov M, Sekulić A, Nektarijevic D, Pantovic S, Šljivančanin D, Dugalic S (Clinical Centre of Serbia); Đermanović A*, Radovanovic Z, Radovanovic D, Đurić M, Zahorjanski S (Oncology Institute of Vojvodina); Milosavljevic V*, Zdravkovic D, Toskovic B, Marjanović U, Zdravkovic M, Petricevic S, Crnokrak B, Maric S, Sekulic A, Colakovic (Ristic) N (University Hospital Medical Center Bezanijska kosa); Kovacevic B*, Krdzic I, Bojičić J, Sparic T, Ostojic A (Zvezdara University Medical Center).

**Slovenia**: Cokan A*, Košir JA*, Pakiž M, Potrč S, Kavčič N, Ivanecz A, Horvat M, Jagric T, Kacjan U, Perić I, Timošek E, Plahuta I, Turk Š, Crnobrnja B, Kovačič R, Dovnik A, Al Mahdawi L, Knez J, Grosek J, Košir Božič T, Tomazic A (University Medical Centre).

**Spain**: Suarez Gonzalez LA*, Busto Suarez S, De La Infiesta García C (Complejo Asistencial Universitario de León); Arencibia O*, Gonzalez Garcia-Cano D, Laseca M, Martin Martinez A, Guijarro Guedes JA, Rave Ramirez AF, Gil Gonzalez Y, Hernández Socorro AM, Ponce Arrocha D, Cruz García A, Mendoza Rodriguez C (Complejo Hospitalario Universitario Insular); Catala E*, Romero Garcia CS, García Trueba A, Georgieva V, Hernández Álvarez AI, Martinez-Perez C, De Valera E, Gascón JJ, Lopez Alcina E, Samper Monton L, Aghababyan K, Valero Tarin A, Salinas Lozano C, Cervera A, Catalá Bauset JC, Ramia B, Marques Peiro F, De Andres J, Iserte Juan SP, Peñalver Gaspar M, Gilabert-Estellés J (Consorcio Hospital General Universitario); De La Rosa-Estadella M*, Fernandez-Colorado A, Serrano-Martin M, Gasulla-Rodriguez A, Juarez-Pomes M (Corporación Sanitaria Parc Taulí); Ossola ME*, Sanz Ortega G, Gómez J, Del Campo M, Corrochano R, Galan Hernandez M, Molano Nogueira FJ, Ibañez Vazquez L, Dziakova J, Zarza Martín A, Infante S (Hospital Clinico San Carlos); Moro-Valdezate D*, Pérez Santiago L, Molina AI, Vinuesa Huesca A, Gadea Mateo R, Navarro-Sanchis L, Aracil-Boigues P (Hospital Clínico Universitario de Valencia); Jezieniecki C*, De Andrés-Asenjo B, Zamora Horcajada Á, Natal Álvarez F, Cuellar Martin LA, Montes-Manrique M, Cabezudo G, Gómez Sanz T, Herranz Arriero A, Sierrasesumaga N, Rodriguez-Lopez M, Ruiz Soriano M (Hospital Clínico Universitario de Valladolid); Sánchez Gollarte A*, Minaya Bravo AM, Guijarro Moreno C, Galvan A, González E, García Ureña MÁ, Robin Valle De Lersundi A, Ruiz-Tovar J (Hospital del Henares); López Campillo A*, Jimenez Toscano M, Salvans S, Jaume Böttcher SM, Godó À, Busquet Raich G, Téllez C, Sánchez García JA, Ribas Ardanuy M, Saavedra P, Sánchez Cabrero A, Clivillé-Pérez J, Durante Escutia M, Rabasa Rodríguez A, Aguilera P (Hospital del Mar); Giménez Francés C*, Ruiz-Marín M, García Escudero D, Valero Soriano M, Ramirez Faraco M, Galán Jiménez S, Alcón Cerro P, Rodríguez Lucas JM, Carrasco Prats M, Peña Ros E, López Sánchez MDP, García Porcel VJ, Tamayo M, Muñoz Camarena JM, Albarracin Garcia R, Molina O, Moreno Sánchez P, Jiménez I, Pastor Perez P, Agea Jiménez MB, Candela Mas D, Artés Artés M, Benavides Buleje JA, González Valverde FM, López-Morales P, Sanchez A, Bobadilla Romero ER (Hospital General Reina Sofía); Allue M*, Ponchietti L, Latorre Tomey R (Hospital General San Jorge); Torres Ramos D*, Jimenez-Gomez LM, Valdés López-Linares S, Moreno Salazar YT, Soto Galán J, Moreno De Alboran C, Prosperi A, Osma JJ, Blanco A, Sánchez Rodríguez M, Valdivielso E, Cejudo Á, Perez Carpio C, Vasques Seabra Águas F, Sánchez Martín E, Pérez-Ajates S (Hospital General Universitario Gregorio Marañón); Macarulla E*, Álvarez Torrado A, Galmés C, Artigot M, Marin Garcia J (Hospital Igualada); Oliver Guillen JR*, López De Fernández A, Fernández-Velilla San-José BF (Hospital Santa Bárbara); Ambrona Zafra D*, Pérez Farré S, Ortega Alcaide J, Codina Corrons L, Sisó Soler E, Gómez Báez FD, López-Soler G, Gutiérrez Pérez E (Hospital universitari Arnau de Vilanova); Aix Molina S*, Vallve-Bernal M, Varona Mancilla A, Aldama Martín ÁM, Casanova R, Sevillano L, Muela JJ, Rodrigo Rodrigo M, Goujon I, Fructuoso Iniesta C (Hospital Universitari De Tarragona Joan XXIII); Pérez-Bertólez S*, Monclus E, Fernández L, Silva Hernández MG, Vicario F (Hospital Universitari Dexeus); Garcia Pimentel P*, García Álvarez R, Bouzó Molina N, Ramiro Á, Calderon Barajas ZA, Sanz R, Xia Z, Rodríguez P, Sarrais Polo C, Martínez López A, Estrada C, Gómez De Castro PE (Hospital Universitario 12 de Octubre); Fernández Martínez D*, García Flórez LJ, De Santiago Alvarez I, García Alonso L, García-Santos G, García González LA, Carrasco B, Silva-Cano DW, Del Val Ruiz P, Martínez Izquierdo G, Corteguera A, Iturbe J, López-Negrete Cueto E, Cembellin A, Ramos Montes C, Ibero Casadiego GP (Hospital Universitario Central de Asturias (HUCA)); Prieto M*, Villalabeitia Ateca I, Pascual Vicente T, Villota Tamayo B, Perfecto A, Mambrilla S (Hospital Universitario Cruces); Ramos Del Moral IL*, Jiménez Carneros V, Pastor-Riquelme P, Franco Lozano A, Alonso-Lamberti L, Lozano M, García-Quijada J, Zhang R, García A, Martín MM, Yeh Ahumada M, Carreño Pallarés A (Hospital Universitario de Getafe); Pérez Rubio Á*, Goran RE, Gómez Diez N, Zomeño Bravo G, Bernal-Sprekelsen JC, Martínez Fernández GS (Hospital Universitario Doctor Peset); Marquina González R* (Hospital Universitario Donostia); Toribio-Vazquez C*, Eguibar A, Perez-Chrzanowska H, Serrano Méndez P, Valderrabano Gonzalez S, Reinoso FJ, Yebes A, Alonso Bartolomé MB, Ayllón Blanco HR, Cristóbal I, Pellicer I, Arenal González R, Zafra Angulo JD (Hospital Universitario la Paz); Duque Mallén V*, Gracia-Roche C, Gascon Ferrer I, Aparicio-López D, Antonio M, Herrero Lopez M, Saudí-Moro S, Gascón Domínguez MÁ, Gimenez Maurel T (Hospital Universitario Miguel Servet); Matías-García B*, Morales Palacios N*, Diez Alonso M, Gutiérrez A, Soto Schütte S, Bru-Aparicio M, Jiménez Martín R, Sanchez Pellejero A, Vázquez Valdés S, Vera Mansilla C, Quiroga A, Mendoza-Moreno F, Diego García L, Serrano Yébenes E, Mañes Jiménez F, Lasa I, Ovejero Merino E, Casalduero L, Allaoua Y, Blazquez Martin A, Córdova García D, Alvarado Hurtado R, Laguna Hernández P, Garcia-Moreno Nisa F, Juara H (Hospital Universitario Principe de Asturias); Aldecoa C*, Tejero-Pintor FJ, García M, Aguado Saster E, Ruiz De Santos E, Bueno Cañones AD, Pérez Fernández S, Alija Garcia MG, Urruchi R, Rioja Garrido R, Madrid M, Bordell A, Arranz Chamorro I, Rodriguez Cañal P, Pacheco Sánchez D, Marcos-Santos P, Veleda Belanche S, Maestro De Castro JL, Blanco García MJ, Laita Jiménez E, Leal L, Vaquero Perez L, Barbosa C, Ramos Carrasco M, Garcia-Saiz I, Rodicio P, Acebes García F, Lizarralde A, Sanchez Diez A (Hospital Universitario Río Hortega); Ferrer-Inaebnit E*, Segura-Sampedro JJ, Alfonso Garcia M, Gutiérrez-Cañadas GGC, Oseira A, Torres Marí N, Loyola J, Villalonga B, Camporro P (Hospital Universitario Son Espases); Pagès N*, Colás-Ruiz E, Cifuentes Rodenas JA, Castro Suárez M, Moll-Amengual R, Mata J, Fernández Manzano J (Hospital Universitario Son Llàtzer); Gómez Pérez B*, Gil Martínez J, Cerezuela Fernández De Palencia Á, Aliaga Rodriguez A, Gómez Valles P, Delegido García A, Gil Gómez E, Montoya MJ, Navarro-Barrios A, Cayuela V, Ruiz Manzanera JJ, Jiménez Mascuñán MI, Gutiérrez Fernández AI, Sánchez Esquer I, Gil Vázquez PJ, Ferreras D, Balaguer Román A, Gómez-Bosch F, Alconchel F (Hospital Universitario Virgen de la Arrixaca); Romero A*, Durán Muñoz-Cruzado VM, García Sánchez CJ, Tejero Rosado A, Ruiz B, Roman MDC, Rubio Fernández JJ, De Jesús Á, Ostos Diaz M, Quintero-Pérez C, Sánchez Marín A, Alvarez Aguilera M, Leal Mérida M, Ager I (Hospital Universitario Virgen del Rocio); Sancho-Muriel J*, Frasson M, Castro B, Guerrero P, Nieto-Sanchez M, Cruz Q, Cholewa H, Hurtado-Pardo L, Plazas D (Hospital Universitario y Politécnico La Fe); Serrano-Navidad M*, Castillo J, Ballestero R, Valbuena Jabares V, García C, Garcia Formoso N, Herrero Blanco E, Suarez Pazos N, Cagigas Fernandez C, Gomez Ruiz M, Benic Yoris YT (Marqués de Valdecilla University Hospital); Merayo M*, Gonzalez Fernandez ME, Alonso Batanero S, Rodríguez Gómez J (Universitary Hospital El Bierzo); Landaluce-Olavarria A*, Estraviz B, Markinez I, Gómez D, Fernández Gómez Cruzado L, González De Miguel M, De Francisco Rios JM, Urigüen A (Urduliz Alfredo Espinosa Hospital); Gómez Del Pulgar A*, Espin-Basany E, Gil-Moreno A, Bellmunt-Montoya S, Dopazo C, González-Suárez S, Blanco-Colino R, Olid Mayoral P, González Antúnez M, Del Barco Martínez E, Bejar Dolcet Y, Gil-Sala D, Marrero Eligio De La Puente C, Sánchez Besalduch L, Barrio Zaragoza M, Lopez Lozano JF, Olaria C, Catalan Sanz S, De Ciria E, Segura Farran L, Montes N, Zanca Gómez JM, Puig Escobar I, Armengol Alsina M, San Nicolas M, Escura M, Sánchez M, Rivas Agudo M, Aalouf L, Maya Salas D, Hernando Marín L, Umpiérrez Mayor N, Sanchez Iglesias JL, Fernández Henriquez Y, Feixa Molina I, Gomez-Jurado MJ, Bebia V (Vall d'Hebron University Hospital).

**Switzerland**: Racine M*, Sauvain M, Mujcinovic A (Hopital de Pourtales); Bolla D*, Dimitrov Y (Hospital of Langenthal); Arenja N*, Riboni C, Marx JN, Kalisvaart M, Andreou C, Brandts P, Pfefferkorn U, Eisner L, Dietz U (Kantonsspital Olten); Gass J*, Metzger J, Scheiwiller A, Kremo V (Luzerner Kantonsspital); Gero D*, Bueter M, M F, Schulz R (Männedorf Hospital); Mongelli F*, Soave I*, La Regina D, Canonica C, Sabbatini F, Gaffuri P, Spampatti S, Santarelli C (Ospedale Regionale di Bellinzona e Valli); Di Giuseppe M*, Marengo M, Passoni S, Dellaferrera G, Provenzi D (Ospedale Regionale di Locarno La Carità); Bernardi L*, Papadia A*, Cristaudi A, Gasparri ML, Aurora P, Popeskou SG, Christian P, Antonilli M, Scalvi FA, Sevas V, Palumbo G, Maoloni L, Cencetti C, Pagnani G, Hitz M, Dürmüller J (Ospedale Regionale di Lugano); Däster S*, Hess GF, Aegerter NLE, Sedlaczek P, Soysal S, Zeindler J, Angehrn F, Müller L, Taha-Mehlitz S, Haak F, Nocera F, Karli T, Lazaridis I, Tampakis A, Lalos A, Friedrich U, Wiesler B, Varathan N, Frey R (University Hospital Basel).

**Turkey**: Kurtoglu GK*, Aghayeva A, Turam C, Kurtoglu GM, Maarouf M, Baca B, Ilhan SD, Koyluoglu YO, Seker ME, Ulufi M, Erkaya M, Kılıçkan FDB, Bayrakceken S, Ozmen BB, Togay B, Doğan M, Çetinkaya T (Acibadem Altunizade Hospital); Tuzuner E*, Karahasanoğlu T, Yavuz IE, Bilgin IA, Kekik SS, Turam C, Dönmez E, Dikeç M, Ramoğlu N, Yurtseven N, Takmaz O, Orhun Ö, Karadeniz N, Saylık O, Uğur EM, Ada E, Durbas A, Hamzaoglu I, Uzun K, Gungor M, Gebedek IY, Hayırlıoğlu EÇ, Halıcı M, Ceyhan GO (Acibadem Maslak Hospital); Mutlu AU*, Aytac E, Gulmez M, Capkinoglu E, Yilmaz TU, Ozer A, Keçeoğlu S, Eraslan ZC, Ozben V, Dülgeroğlu O, Saraçoğlu C, Erkan M, Uras C (Acıbadem Mehmet Ali Aydınlar Üniversitesi Atakent Hastanesi); Copur HD*, Dogan NU, Topal E, Demirsoy I, Korkmaz D (Akdeniz University Hospital); Can U*, Buğra D, Sobutay E, Çakıt H, Ergün E, Zenger S, Bilgic C, Gurbuz B (American Hospital); Sünter K*, Gecim I, Sefer S, Inceöz B, Genc MB, Uygun AK, Yücel E (Ankara University Hospital); Barcin A*, Altinel Y, Aktimur YE, Meriç S, Özdoğan K, Sayar AI, Durmaz AG, Çakır I (Bagcilar Research And Training Hospital); Ünlü ES*, Kiran G, Dal A, Ozkan E, Kalkan S, Öncel MM, Çetin Ç, Yılmaz G, Karimi H, Iskurt Y, Yardimci E, Eker M, Balkan AO, Gönül HB, Herdan E, Ertugrul M, Takmaz T, Kilinç SS, Pinar MS, Yıldıztekin F, Coşkun EC, Olgun I (Bezmialem Vakif University Hospital); Ince G*, Isik O, Şen M, Özata R, Eroğlu B, Büyükpolat B, Ishakoğlu G, Özen D, Aktaş MO, Kılıç B, Yılmazlar T, Koçbey MS, Işıklı N, Yıldız RG, Baghirov T, Uygun E, Yugaç M, Keskin E (Bursa Uludag University School of Medicine); Bozkurt BE*, Tas S, Türkmen ÖÖ, Ata B, Alan B, Işık N (Canakkale Onsekiz Mart University Hospital); Göver T*, Bisgin T, Özalp E, Sökmen S, Manoğlu B (Dokuz Eylul Univ. Hospital); Bakar C*, Bozbiyik O, Zorlu U, Zengin NN, Baki BE, Akbulut ES, Tunali S, Kutlu B, Oğuz YA, Küçükateş B, Yildiz E, Sakmar AN, Yoldas T, Akgün ZE, Korkut MA, Çalışkan C, Aksit E, Aksalman EF, Koçoğlu AO, Basci F (Ege University Hospital); Yigit B*, Ilhan O, Liman RK, Uzun M, Ağaçkıran I (Elazig Fethi Sekin City Hospital); Şahin C*, Leventoğlu S, Kubat Ö, Genç I, Oyanik A, Gurbuzer FI, Satilmis N, Cam S, Ekinci M, Gönül M, Chasan D, Zara SGF, Sök Ş, Yalcinkaya A, Acıpınar ET, Timurçin U, Afzal NB, Gillani SHA, Gillani S (Gazi University Medical Faculty Hospital); Yazıcı S*, Cennet O, Suer MS, Kaya Ö, Uslu EZ, Tanrisever A, Yildirim C, Gunenc IN, Gül B, Özkal I, Tileklioglu HI, Korkmaz MA, Şirin I, Özbayrak U, Mıdık A, Domaç E, Karakoç A, Urek S (Hacettepe university hospital); Yıldırım A*, Ugur M, Doğru M, Arslan ER, Ular B, Duymuş ME, Turhan AB (Hatay Mustafa Kemal University Training and Research Hospital); Dogan T*, Ozkan MB, Benek DE, Arslangilay M, Yildirim MB, Yazgan Y, Tıraş HN, Şahin BI, Çapar MA (Hitit University Faculty of Medicine Hospital); Tatar C*, Arı A, Batikan O, Güler M, Naycı AE, Sevinc MM, Şevik H, Oğuz C, Karagülle OO, Idiz UO, Demir A, Çakır E, Cakir C, Doğan S, Yıldız I, Gürlük M, Cengiz MB, Toptaş M, Akay O, Kaya R, Aydin E (Istanbul Education and Research Hospital); Çakıcı MÇ*, Yildirim A, Efiloğlu Ö, Demirtaş B, Iplikçi A, Atis G, Keser F, Culpan M, Çiçek M, Tahra A, Akalin MK (Istanbul Medeniyet University Hospital); Izgiş A*, Soytas M, Okkabaz N, Saylar A, Onaran E, Askin AE, Hashimov E, Bektas S, Sabuncu K, Alagöz AC (Istanbul Medipol University Hospital); Karatas I*, Gok AFK, Simsek E, Altunay ZI, Koç E, Iscan Y, Sormaz IC (Istanbul School of Medicine); Yabaci BI*, Ertürk MS, Nazli M, Saglam IE, Erginöz E, Ozcelik MF, Gokden M, Guler C, Gunaydin FI, Uludağ SS, Ibis B, Bozkir HO, Karaca MB, Ulucay MC, Oner KB, Akbas S, Calikusu FZ, Akcan H, Ugural S, Zengin AK, Ozdemir Z (Istanbul University- Cerrahpasa, Cerrahpasa Faculty of Medicine); Demirbas I*, Guner A, Tepe MD, Kıralı N, Karabağ BC, Pehlivan D, Yuzgec AN, Yıldız B, Akin B, Aktaş M, Karabulut E, Cepe H, Küçükaslan H, Salih SS, Uzun M, Yücesan Ö, Reis ME, Ulusahin M, Aşcioğlu K, Tufan E (Karadeniz Technical University Farabi Hospital); Saleem Q*, Saracoglu K, Ozbilen M, Kale A, Geçici ME, Bulun Y (Kartal Dr. Lutfi Kirdar Training and Research Hospital); Uysal Ö*, Atay A, Şuataman B, Guducu C, Çinar TN, Kul H, Canpolat D, Ipekoğlu M, Aydemir M, Karahan F, Hizli K, Kaya B, Yenen OC, Gümüş B, Turmuş K, Vatansever S, Candan I (Katip Celebi University Faculty of Medicine Hospital); Sucu S*, Balik E, Bozkurt E, Özata IH, Ozoran E, Tüfekçi T, Karahan SN, Sarıkaya AF, Agcaoglu O, Danacı S (Koç University Medical School); Ulutaş ME*, Hasirci I, Arslan K, Metin ŞH, Yılmaz A, Turan E, Şimşek G, Kılınç A, Şahin ME, Ozden S, Bayraktar YA, Maden AS, Şahin A, Acar N, Erşen O, Balcı E, Yaldız H (Konya City Hospital); Guldogan CE*, Ozmen MM, Gundogdu E, Moran M (Liv Hospital Ankara); Celik B*, Sivrikoz E, Karabulut K, Dural AC (Liv Hospital Vadistanbul); Ikizoğlu T*, Aydede H, Yalçın T, Dalkıran A (Manisa Celal Bayar Üniversitesi Hafsa Sultan Hastanesi); Yapici HB*, Uprak TK, Tatar Y, Aksahin Y, Aral C, Cetin SZ, Artvin D, Tatar Z, Karakuş IS, Çatal S, Nalbantoğlu B, Bettar S, Düzyol Z, Demir BC, Kırcalı MF, Taşcı ME, Secil F (Marmara University School of Medicine); Akkaya EN*, Kayılıoğlu I, Hekmatjou B, Çağlayan A, Dadaşoğlu MA, Fergar C, Beden MS, Kayhan E, Arslan I, Koçyiğit GZ, Beyoğlu B (Mugla Training and Research Hospital); Varan C*, Sungurtekin U, Sungurtekin H, Özgen U (Pamukkale University School of Medicine); Çetin B*, Pergel A, Uyan M, Erata E, Aldhahebi E, Aslan B, Kabalı Ş, Köse F, Keşaplı I (Recep Tayyip Erdoğan Üniversitesi Eğitim ve Araştırma Hastanesi); Dulger UC*, Altintoprak F, Akçay M, Cünük ES, Kamburoğlu MB, Küçük IF, Yigit M, Sabuncu E, Harmantepe AT, Aka BU, Baş E, Yavuz Akça T (Sakarya Faculty Of Medicine); Sarı AC*, Colak E, Candan M, Kara ME, Ciftci AB, Yemez K, Uyanik MS, Polat S, Kucuk GO, Ocak S (Samsun Training and Research Hospital); Tosun Y*, Unal E, Ofluoğlu CB, Üstbaş C, Gangal Ö (Sehit Prof.Dr. Ilhan Varank Training and Research Hospital); Sanli AN*, Sayır S, Karslioglu C, Ulusoy I (Silivri State Hospital); Tufan AE*, Demir U, Celayir MF, Baran E, Aksoy AB, Fırat DB, Yüksel A, Güven O, Ertaş I, Kuzu ZS, Köksal HM, Gok M, Görgün MN, Yazici P, Ömeroğlu S, Özdemir BB, Kabul Gürbulak E (Sisli Hamidiye Etfal Training and Research Hospital); Ajredini M*, Cakcak IE, Atakan IH, Göztepe A, Budak O, Aydoğdu EO, Erdem E, Yılmaz C, Özdemir J, Akin B, Alkan D, Cengiz E, Donmez CN, Akyüz E, Ergun YH, Ozer MG, Ak Ç, Bolat F, Kasapoğlu E, Tatlisu A, Yorulmaz Y, Enez A, Ozkan B, Yilmaz ZE, Altin SN, Gunes B, Kaban B, Korkmaz O, Demir IB, Öz GA, Tuluce AB, Turkmen ZN, Yildiz F, Kandil B, Ulkucu A, Kıral G (Trakya Üniversitesi Hastanesi); Yavuz D*, Köksoy ÜC, Kus ZC, Erten MB, Mutlu M, Hökelekli F, Aslan T, Orçan Akbuz Ş, Durmuş E, Gökçek TE (Ufuk Üniversitesi Dr. Rıdvan Ege Hastanesi); Ulgur HS*, Ozkan OF, Karakullukcu HK, Yıldız A, Ceyhan M, Çelik MŞ, Kalın M, Kirkan EF, Demirbilek A, Çelik MN, Karbuş Ö (University of Health Science Umraniye Education and Research Hospital); Demirli Atici S*, Abud B, Erdinç H (University of Health Sciences Tepecik Training and Research Hospital); Ön KB*, Sevinç B, Damburacı N, Altiner E, Özdemir B, Manisalı Ö, Ural G, Topal MK, Can T, Ercan S, Kaynar S (Usak University Medical School); Emanet O*, Javadov M, Demir UB, Erdem A, Demircan F, Yelmenoğlu M, Seçen E, Alkışer B, Arık F, Kaya EE, Akkad D, Karaçam D, Karacam U, Yücel MA, Hatipoğlu A, Mumcu N, Karima O, Dal MB, Aljobbeh M, Montaser J (Yeditepe University Hospital); Kara MA*, Gultekin FA, Deniz O, Kavak N, Kum B, Ecir MM, Yavuzer YG, Sezgin Z, Koçdemir U, Yirmibeş B, Atalay Ş, May A, Varna S, Aydoğdu I (Zonguldak Bulent Ecevit University School of Medicine Research and Training Hospital).

**United Kingdom**: Abdel-Fattah A*, Ramsay G, Bin Badekruzaman MA, Karvelyte M, Siamisang M, Clunie S, Mikalauskaite M, Nessa A, Joshi C, Jodeh N, Zafar R, Miller J, Basharat R, Singh T, Weston O, Loy R, Tsoi EHC, Chandiramani A, Sumarlie RJ, Mitchell L, Yousif M, Qadir M, Gannon M, Tong EYH, Tsang J, Elmarghani M, Kaczmarek J, Luangboriboon J, Mcewen R, Tse B, Wong MC, Wong ZX, Diffley T, Jaruwattanapradid O, Ng N, Mchaffie L, Wright C, Joyce D, May V, Emmanuel S, D'costa A, Kumar A (Aberdeen Royal Infirmary); Hanganu C*, Gourgiotis S, Townson A, Williams CY, Baines E, Smith L, Elks N, Simon N, Chintapalli R, Banerjee A, Bhogle S, Ryan B, Maghsoudi D, Clifton E, Nott T, Vickers H, Stewart GD, Zhong ZX, Ioannou K, Ne CKH, Conci R, Fitzsimons-West E, Ling XL, Patel R, Sanghera R, Dokubo I, Seago I, Fairhurst O, Amin B, Aniq M, O’keeffe E, James S, Chowaniec M, Rutigliani L, Hu M, Ferreira AF, Kalogeropoulou M, Hui G, Coakham L, Healy S, Gilanliogullari K, Nishimura G (Addenbrooke's Hospital); Choi M*, Lunevicius R, Aje D (Aintree University Hospital); Mcnicholas R*, Newman C, Bollen E, Kumar A, Ferreira Xavier I, Baloch LG, Koduah-Sarpong NA, O'sullivan G, Saseendran S, Bin Sahl A, Murphy E, Hussain H, Nicholls A (Airedale General Hospital); O'dolan S*, Gidwani A, Patterson E, Loughrey L, Donaghy K, Hana K, Monaghan M, Ni Shandair S, Kulasingha N, Gormley N, Mcglinchey M, Dunlop E, Dunwoody B, Lian SH, Jayasangaran SC, Logue E, Goswami P (Altnagelvin Area Hospital); Ann Jacob P*, O'kane C, Ab Razak AS, Govender K, Mccann EJ, Perry S, Mckendry S, Pachchigar A (Antrim Area Hospital); Singh A*, Dindyal S, Pendyala S, Venkateswaran V, Alhoussan L, Feil F (Basildon University Hospital); Hughes T*, Gerçek Y, Choudry M, Haidar O, Rujeedawa T, Speirs T (Bedford Hospital); Odendaal J*, Chu J, Shahab A, Ranjithkumar S (Birmingham Women's Hospital); Pillai Rajendra Prasad V*, Linn T, Clark J (Blackpool Victoria Hospital); Sharma M*, Lockwood S, Chawla S, Deshpande R (Bradford Royal Infirmary); Long N*, Rees J, Kobetic M, Fawcett-Till D, Ferris O, Harrison G, Hibbs T, Tsang CTW, Hurley L, Jichi T, Badrinath C, Applebee L, Ecott K, Sullivan T, Piecha B, Jagadish A, Sajjad Z, Griggs R, Joyce S, Spurgeon R, Ali Khan M (Bristol Royal Infirmary); Hobrok M*, Roberts R, Mckenna J, Davies D (Bronglais Hospital); Eaton P*, Bishop L, Magaway S, Denholm K, Doyle S, Deshpande R, Salter M (Calderdale Royal Hospital); Chan V*, Mockford K, Heinz J, Ahmed I, Coverdale M (Castle Hill Hospital); McKillen RJ*, Maguire M, Mclees N, Lau J, Mcguigan L, O'Neill A, O'Hare D, Beggs C, Owolabi O, Hunter J (Causeway Hospital); Kinross J, Kotecha J, Doherty R, Patel V, Wagner E, Hodges L, Hassan H, Sribaskaran K, Pouris K, Jain S, Kim AJ, Gill S, Nand S, Toutouza O, Joshi A, Thornley L, Chen R, Ogunjimi M, Mahmood A, Chong R, Yanai M, Bae D, Dhaliwal J, Lovejoy A, Akhbari AR (Charing Cross Hospital); Mayor N*, Kontovounisios C, Yap SHK, Damarla M, Ooi B, Ng MA, Vargas Zhang A, Limbu S, Nyamakope K, Agarwala Y, Zingas N, Cleasby C, Smyth E, Drmota J, Yang QZC, Vedi C, Samarendra S, Nayak V, Rajendra S, Choudhry M, De Stadler K (Chelsea and Westminster Hospital); Bandyopadhyay S*, Bond-Smith G, Collart O, Baffour-Awuah K, Shah R, Mcnamara J, Tadikamalla G, Wilson B, Cossins C, Li H, Ismail L, Soleymani Majd H, Chang E, Jallow A, Baldelli S, Alberti P, Grant O, Doody M, Borawska Z, Bowman A, Clay H, Petit Y, Kimani JG, James M, Hussain SF, Nezhentsev A, Emmerson M, Vijjhalwar R, Shaw G, Holmes C, Ying Y, Farache Trajano L, Anis A, Dequaire J, Hunter A, Danvers R, El-Nemr M, Hammett C, Pikoula M, Thompson S, Lander B, Dierksmeier S, Sadeghi N, Suribhatla R, Ahmed R, Pandit S, Thornton W, Thornton-Swan T, Johal A, Khan M, More A, Tilahun Y, Hauperich A, Gidda R, Vorley I (Churchill Hospital); Khan Z*, Sintler M, Van Den Broeck A (City Hospital); Georgiou M*, Eardley N, Salem H, Yemparala S, Whittle L, Jagger C, Turner K, Vernon L, Aldred K, Manokar P, Moore A, Mistry N, Zgliczyńska J, Haylett C, Adeyanju A, Headford E, Khanna R, Deef M, Durkin T, Trayling E, Cowell R, Khan Z, Turner A, Noureldin K, Down W, Cyril A, O'halloran C (Countess of Chester Hospital); Shirke M*, Epanomeritakis M, Karnati A, Sreejith G, Sinton C, Oliver L, Ali Azamatullah Khan HF, O’kane E, Trouton J, French B, Campbell I, Curran R, Brines C, Mcdermott A (Craigavon Area Hospital); Yang N*, Vig S, Chowdhury S, Valecha R, Lau R, Nafis M, Thavanesan S, Marks J, Akaje-Macauley L, Patel N, Beard E, Nanda A, Mills C, Cheung A, Amalendran J (Croydon Hospital); Todd L*, Smolarek S, Warrener T, Sen S, Mudehwe A, Khan S, Wisden B, Lau G, Schanzer L, King Z, Giridhar K, Reed-Embleton M, White L, Filippidis P, Lee-Smith L, Carless O, King C, Herriott M, Saju N, Tin Maung YM, Deligianni A, Mumtaz A, Khan S, Hill H, Sahdev S, Thomas I, Shah A, Saji S, Hargreaves J, Khan R, Tharani A, Ullah R, Ludgate J (Derriford Hospital); Shrestha S*, Bota E, Abdullah A, Allison M, Patel Z, Clark A (Diana, Princess Of Wales Hospital); Suchett-Kaye I*, Stubbs B, Holmes B, Santaniello A, Watkinson T, Crimmins A, Gupta MK, Abraham S, Bellringer A, Clowes R, Chatzopoulou D, Kiran A, Eid J, Rao N (Dorset County Hospital, Dorchester); Caterson J*, Soggiu F, Malik H, Jose I, Kavallieros K, Rizk C, Thummala B, Li K, Goel A, Pantula R, Toh J, Fu MX, Ho CC, Vivek K (Ealing Hospital); Owen E*, Day A, Jamieson A, Hassani H, Thavarajah T, Vivekanandan M, Muneer A (East Surrey Hospital (Redhill)); Chauhan M*, Veeramootoo D, Ali S, Dosanjh J, Newing F, Jose E, Chauhan H, Kulshreshtha A (Frimley Park hospital); Thomas E*, Patel P, Chhabra R, Sajan B, Ragavan S, Hariharan R, Lam I, Fleet B, O'hara N, Wright A, Reddy V, Darweesh H, Khan A, Handa S, Kewada D, Rana F, Williams H, Bombieri F, Shah N, Pestotnik Stres D (Furness General Hospital); Menon A*, Selvam L, Pusok A, Street C, Zohdy M, Aguirrezabala Armbruster B, Okoye I, Potts L, Fayyad M, Bajaj K, Alfa H, Sivakumar N (George Eliot Hospital); Duncan N*, Roxburgh C, Huang L, Lukito K, Huang X, Baig S, Chan CY, Philip R, Shaheen L, Dodds J, Yeow HX, Devindran M, Sia ZC, Park JH, Furze B (Glasgow Royal Infirmary); Yung N*, Vipond M, Jones M, Asekun F, Dembinska-Kenner A, Saleh R, Larkai E, Harris J, Cherian M, Louden H, Bisbinas V, Behl A, Hughes R, Smith R, Zaman Z, Hoskyns O (Gloucestershire Royal Hospital); Carlton H*, Thorn C, Tourky M, Tan J, Sen K, Elsey J, Bevan J, Ko N, Kalidindi S (Great Western Hospital, Swindon); Rooney M*, El-Boghdadly K, Zafar S, Wyncoll A, Morillon P, Sennaraj M, Mahfouz H, Afzal A, Sibal R, Suji T, Headon H, Abdul A, Ahmed S, Mcnally-Reilly JP, Omran K, Mulcahy C, Aftab M, Haghighat Ghahfarokhi M, Farhangi M, To WY, Ho M, Patel T, Siu A (Guys and St.Thomas' Hospital); Choudhry H*, Huntley M, Hussain Z, Wong G, Maguire R, Khan M, Potts A, Ahmed M, Ramesh G, Kolade O (Harrogate District Hospital); Siddique MH*, Griffiths E, Qureshi M, Hoque M (Heartlands Hospital, Birmingham); Laulloo S*, Das K, Waheed N, Tang TYT, Ahmed R, Habib R (Hereford County Hospital); Vyas R*, Watson S, Theodoropoulou K, Siu M, Yu N, Islam N, Burnett J, Besso-Cowan A, Hirani D, Zsolnai I, Chen YK, Dupere O, Keitley L, Park C, Verma I, Chan J, Tavner J, Nicolaou M, Lee NYS, Hegy R, Lee RBY, Yi Lun KYL, Reyes D, Dong S, Thornton J (Homerton Hospital); Eaton P*, Bishop L, Magaway S, Denholm K, Doyle S, Deshpande R, Salter M, Weir M, Gibbs A (Huddersfield Royal Infirmary); Al-Shaye A*, Alwahid M, Tait A, Smith S, Doye A (Inverclyde Royal Hospital); Law KL*, Groot-Wassink TG, Geller G, Seebah K, Cox O, Kalogeropoulou M, Shetty A, Oh DCJ, Lee E, Packham B, Aarons M, Saadeh K, Mui J (Ipswich Hospital); Huynh R*, Eid M, Honey N, Kaur J, Hand R, Lai L, Koubaesh C, Maqsood-Shah H, Suresh J (James Cook University Hospital); Collart O*, Bond-Smith G, Shah R, Mcnamara J, Tadikamalla G, Wilson B, Cossins C, Li H, Soleymani Majd H, John L, Chang E, Kimani JG, Jallow A, Bandyopadhyay S, Baldelli S, Grant O, Doody M, Borawska Z, Bowman A, Foster C, Clay H, Petit Y, James M, Hussain SF, Nezhentsev A, Emmerson M, Vijjhalwar R, Shaw G, Holmes C, Ying Y, Farache Trajano L, Anis A, Dequaire J, Hunter A, Danvers R, El-Nemr M, Hammett C, Pikoula M, Thompson S, Shah R, Dierksmeier S, Sadeghi N, Lander B, Suribhatla R, Ahmed R, Pandit S, Thornton W, Thornton-Swan T, Johal A, Khan M, More A, Tilahun Y, Hauperich A, Gidda R, Vorley I, Lewis-Orr E (John Radcliffe Hospital); Reilly S*, Bhatia A, Goves K, Blesson M, Purser L, Cobb L, Sarker MH, De Sousa N (Kettering General Hospital); Syed S*, Rajendran N, Tan Sue Wei J, Navakumar G, Butnari V, Senthilkumar M, Chew ZQ, Zekaite L, Paranietharan S, Haque M, Balenthiran S, Chin KJ, Ahuja S, Moothathamby T, Moniati F, Kong YZ, Lee EJH (King George Hospital); Sharma L*, Found P, Sivakumaar Y, Senior H, Simeen N, Arora A, Chu A, Mizori R, Beazer T, Patel N, Dudziak JM, Squeri T, Weston F, Zhou M, Van Ross J, Tian S, Bedford J, Lam R, Karami Tireh Shabankareh F, Donkin Everton H, Wilson K (King's College Hospital); Richens A*, Bragg D, Akbari A, Awodiya A, Osula SA (King's Mill Hospital); Gerges F*, Gerogiannis I, Daniels L, Seth S, Baxter Z, Nour E, Fitchford L, Perrott C, Abuelgasim M, Flanagan J, Gillani S, Lewis H, Dunscombe O, Steven Goodwin Moughton P, Mahmood A, Hirniak J, Moses A, Kapsampelis P (Kingston Hospital); Ahmed A*, Burke D, Anderton C, Lee E, Conley M, Khan L, Surti R, Waseem F, Sharma M, Imran A (Leeds General Infirmary); Motiwale R*, Singh B, D Sa S, Charuvila S, Lorgat MZ, Chowdhury R, Gogineni P, Finney A, Tzortzi De Paz A, Panesar S, Sidki A, Warwick C, Kadambande S, Patel J, Rao A, Sharma A, Ramewal S (Leicester General Hospital); Ramesh P*, Norwood M, Mehta P, Bhatia M, Tiwana S, Foote E, Rushton F, Asi S, Notaney R, Sinha R (Leicester Royal Infirmary); Vibhishanan J*, Gupta S, Conci R, Chintapalli R (Lister Hospital); Ahmed S*, Sagar J, Ragge N, Rizvi SS, Ashraf M, Cirocchi N, West R, Obiri-Darko E, Rai Y, Hussain S, Ul Ain N (Luton & Dunstable University Hospital); Amir FN*, Smart C, Melomud R, Saad D, Bengeri S, Kuna S, Chaudhary M, Olanite OG, Khan F, Nausheen S, Dodds R (Macclesfield District General Hospital); Boyden H*, Donnelly D, Adegboye O, Osborne L, El-Barraj S, Shukla J, Raza N, Khalid A, Agrawal D, Kyaw KT, Borhan S, Pangrazi G, Ioannou I, Ahmed AN, Savvides G, White M, Puthur SJ (Manchester Royal Infirmary); Bridgewater S*, Waraich N, Bryce R, Khosla R, Penhaligan L, Desai A, Ehsan Z, Mostafa O (Manor Hospital, Walsall); Kamel Y*, Keeler B, Rajivan R, Omar V, Gamadia S, Rana D, Srikumar S, Heidari A, Ahsan Akhtar A (Milton Keynes University Hospital); Tarafdar A*, Barry J, Davies D, Curr A, Jimenez-Reid I, Mckenna J, Garry E, Lallmahomed N, O'leary K, Barker E, Jones DR, Tabatabai R, Garg J, Henshaw E, Abdulshafea M, Prabaharan S (Morriston Hospital); Wiles H*, Bamford R, Blest F, Acquah L, Simpson H, Guerrero Enriquez J, Pringle H, Shahid M, Wharf O, Kumar M, Buadooh K, Hanson M, Mutanga I, Hardy A, Usman H, Shams S (Musgrove Park Hospital, Taunton); Schottler N*, Garg A, Sarodaya V, Ali S, Cullen I, Pregil L, Fernandes Silva Ramos M, Hao Y, Das Chagas E Silva AC, Sellahewa S, Li J, Cox J, Sinha P, Lee IJJ, Voniati E, Phillips H, Mooney O, Sockett M (Newham University Hospital); Patil P, Elsllabi M, Chan WKM, Grimble L, Richardson H, Johnston A, Kouli T, De Sousa JFM, Goh YK, Grant C, Martin L, Peh ZH, Yap T, Tang ETW, Lai HYM, Berry T, Rokia M, Tennant E, Lee I, Rahman MJ, Kamal A, Ali S, Hau HX, Harten I, Eloofy M, Caldwell C, Leckie R, Tasnim F, Ravintharan K, Mcauley J, Cameron F, Veerni RB, Looi KY, Ismaili A, Gresz R, Chong V, Tan LK, Singh K, Ashburn S, Shantha Kumar R, Kamal M, Kamal H (Ninewells Hospital); Aziz I*, Stather P, Mylvaganam L, Deliyannis E, Tompkins B, Sikorski E, Harris F, Sanders I (Norfolk and Norwich University Hospital); Fakhrul-Aldeen M*, Cross K, Tahir A, Yim P, Mayne I, Mahapatra S (North Devon District Hospital); Ng CE*, Ortega A, Supparamaniam S, Bowman M, Sadler M, Rogger M (North Durham University Hospital); Gendia A*, Ahmed J, Sivakumaran P, Brick C, Ansong E, Ha H, Sharma A, Wilson N, Manavi B, Zeze L, Gupta NP, Al-Hassani S, Williams C, Ramesh M, Shukla B (Northampton General Hospital); Drye E, Hussain H, Ghatauray E, Tan ACW, Adamu-Biu F, Arora J, Mcallister T, Fairclough S, Economou A, Utulu S, Fung KW, Epanomeritakis IE, Zaman SS, Olszewski E, Ballesteros P, Okpii EC (Peterborough City Hospital); Walkeden E*, Macklin C, Jamil S, Zhen S, Ahmed O, Saravana Kumar A, Uddin H, Hakim R, Mossanen Parsi M, Awoloto O, Rowe K, Mashadi R, Mohammed Z, Chong LC, Fairlie-Vogt S, Russell O, Ozarek W, Little A, O'farrell C, Miller R (Pinderfields Hospital); Froud J*, Thippeswamy K, Thomas R, Fitzpatrick A, Yoganathan S, Dunlop M, Ngai K, Liddell-Lowe T, Barrow A (Prince Charles Hospital, Merthyr); Sharma L*, Found P, Mizori R, Rabas S, Al-Housni R, Zhou M (Princess Royal University Hospital); Marton A*, Chen P, Hasan H, Chan CFB, Win Mar WW (Queen Elizabeth Hospital King's Lynn); Kisiel A*, Griffiths E, Yusuf M, Sinha A, Blenkinsop J, Rawlings K, Chaudri T, Westland G, Umar H, Lee S, Cherian J, Gurhan Z, Chaudri T, Sheraz MS (Queen Elizabeth Hospital, Birmingham); Khan DMI*, Kucukmetin A, Malone D, Betts C, Ali M, Giblin M, Nowicki L, Korompelis P, Farley O, Fouweather M, Ioannides C, Yilmaz H, Landais R, Ngeyu EX, Erinjeri C (Queen Elizabeth Hospital, Gateshead); Badran D*, Glen P, Aamir J, Aziz A, Su PL, Choong I, Reece M, Kulasekaran A, Tengku Saifudin TNS, Luckhurst J, O'hare R, Smith R, Siu YT, Kwok PH, Sood V, Imran A, Fatima K, Munir Z (Queen Elizabeth University Hospital, Glasgow); Kisova T*, Rajendran N, Islam F, Teehan E, Sadik H, Patterson U, Rahman N, Harrisrhaj H, Govardhan D, Rajesh S, Mumtaz Z, Ghori OS, Nawaz H, Lysomirski A, Pikoula V, Qadir RMAB, Jones A, Chiang Y, Morsy H, Pazaiti C, Asardag AN, Ali I, Althonayan J, Badri A, Evans J, Chung E, Harriss L (Queens Hospital); Sinan L*, Maxwell-Armstrong C, De A, Altman R, Varghese F, Chua W, Ahmed S, Javed M, Aljubure R (Queens Medical Centre, Nottingham); Bradley A*, Moug S, Kemmett OEH, Underwood I, Fitzpatrick I, Wong LS, Vakeesan B, Potter K (Royal Alexandra Hospital, Paisley); Varghese T*, Daren S, Hazrin Fazail A, Farajzadeh Asl S, Nautiyal H, Saadeh A, Vial I, Karimi R, Akthar A, Egiz A, Masood R, Qadri A, Chawla T, Yoon H (Royal Blackburn Teaching Hospital); Liu X*, Battersby N, Davies E, Chhetri R, Mclean R, Leaper E, Taylor C, Nicolaisen C, Cochetti L, Zoumi EA, Annable D, Thomas H, James-Knights Z, Lee-Kim-Koon C, Akpinar E, Coelho MJ, Darke E, Morris T, Mayer F, Forbes J, Gold S, Sinha H, Batchelor E (Royal Cornwall Hospital); White L*, Lund J, Forrest S, Dial H, Chishty F, Oliver M, Meyer J, Gokhare Viswanath N, Ammar A, Lopez A, Minhas N (Royal Derby Hospital); Sunny N*, Haynes K, Alexiadis A, Doski S, Arbuthnott R, Memon R, Bukhashem K, Chaudhary A, Sheik-Ali A, Sebastiao A, Taylor C, Clarke E, Misztal S, Raja D, Chui SK, Khalil I, Metsel A, Whelan M, Forrester S, Oboh O, Bayley-Skinner B, Eastwood R, Akapo E (Royal Devon and Exeter Hospital); Jamshaid M*, Osborn G, Woods R, Wooler O, Chumley S, Cotton E, Jarrett N, Amanda N, Johnson J, Quhill M, Al-Ani M, Kandanearachchi S, Khan S, Barrow A, Dunlop M (Royal Glamorgan Hospital); Williams G, Phillips S, Faryad F, Hughes A (Royal Gwent Hospital, Newport); Wong Ching Hwai S*, Johnston C, Guest R, Kaddouri A, Ernst L, Szasz A, Gafrey C, Loy G, Roberts E, Baker S, Davies A, Khan Z, Leonidou M, Chow JK, Murugan R, Baker A, Burns R, Foo YC, Sikora F, Chong L, Chan Ah Song C, Cosgrove C, Reeve-Chen O, Ma J, Mccolm L, Zhang MK, Hussain F, Ng XY, Ingabire B, Wagner B, Bain T, Kovacs R, Small E, Lam S, Yao L, Ho L, G Paremes Sivam H, Dahal K, Stanley A, White M, Hayois L, Thillai M (Royal Infirmary of Edinburgh); Tay T*, Davies E, Kamaruzaman N, Robinson L, Sherrington R, Abid Sohail Z, Lawrence S, Sohail A, Brown D, Parker S, Ohr M, Jasniak D, Crowther B, Jadoon F, Melo LJ, Amin H (Royal Lancaster Infirmary); Lim RQH*, Thaha MA, Bath M, Saadqain SA, Benallal Y, Jin Z, Jackson A, Kuri A, Malik M, Jos H, Goh ET, Paraskevopoulou ME, Kythreotis A, Naim Ahmed Y, Glynou SP, Rehan H, Pereira Pai A, Georgiannakis A, Dworschak N, Cho S, Chow BJ, Khan Y, Enthoven T, Malik A, Latif I, Lakhani AH, Rahman N, Morgan K, Gkolia S, Khalessi N, May PLZ, Lwin A, Kanesaratnam P, Tudgee M, English R, Hu K, Pathanchaly N, Chien DCS, Holloway M, Gao K, Lekka G, Srinivasan R, Zafar S (Royal London Hospital); Peris MR*, Keiarash K, Kanesan H, Chong B, Kobus M, Dominic D, Do DA, Dennis D, Frankland S (Royal Preston Hospital); Shah V*, Youssef B, Sadia U, Hassouneh A, Jaipersad A, Chen T, Fulford M, Dawson A, Kapur K, Jha T, Zope P, Wilkinson M, Bhatnagar V, Knight J (Royal Stoke University Hospital); Madhuri TK*, Katsiari T, Pasternak-Albert N, Moussa E (Royal Surrey Hospital, Guildford); Emms L*, Lamah M, Batool Z, Sabesan A, Islam S, Baig M, Dave M, Liu X, Stuart L, Harvey S, Dam V, Ezuma P, Lee J, Suntharalingam S, Singh S, Preston P, Rickard F, Gupta T (Royal Sussex County Hospital); Madden S*, Jones C, Gidwani R, Wang X, Mcconnell M, Hanna S, Mcgettigan A, Brown K, Cios A, Ward-Bradley C, Hurrell O, Paice V, Mutsonziwa N, Pogoson S, Coulter R, Corry J, Mcclean L, Keenan F, O'dolan S (Royal Victoria Hospital, Belfast); Plonkowski A*, Brady R (Royal Victoria Infirmary, Newcastle); Ooi R*, Iles D, Guillemot LS, Low C (Saint Marys Hospital); El-Barraj S*, Epstein J, Pressler M, Ioannou I, Soliman N, Ktayen A, Macconnachie A, Menendez Lorenzo J, Tooke J, Noman K, Wilkins L, Warner-Levy JJ (Salford Royal Infirmary); Evans R*, Torrance A, Fear C, Vidis Humphries H, Velayudham G, Jefferson-Pillai Z, Brady J, Oza R, Singh D, Hamid A, Hartop P, Shojaie P, Mano J, Rajah T, Cabdi YMC, Singal A, Afzali M (Sandwell General Hospital); Lee JM*, Bandyopadhyay D, Bennett J, Mendenhall P, Millett A (Scarborough General Hospital); Davidson S*, Kallam R, Pishia M, Butler H, Isidore S, Boutsias D, Bryce K (Scunthorpe General Hospital); De Rancourt T*, Barry J, Henry H, Aspinall A, Smith L, Conway C, Shanmugathas H, Huang C, Doherty M, Ahmed K, Chung T, Benson T, Habiba A, Singh Bhangu J, Bingor B, Fice L, Premanandhan H, Mckenna J, Jimenez-Reid I (Singleton Hospital); Hoque M*, Griffiths E, Loughran J, Laskowski M, Mehmood A (Solihull Hospital); O'kane R*, Mullan M, Mcclenaghan R, Kettyle G, Hosty-Blaney F, Chan XHF, Sunny S, Kaur M, Vose S, George V, Killoran D, Doherty R (South West Acute Hospital); Zagorac Z*, Pullyblank A, Ismail R, Anderton N, Dwyer-Joyce L, Morgan R, Zhang N, French B, Cox H, Tetro J, Clarke A, Shaw K, Iau R, King E, Patel S, Ovakimian M, Zahid A, Rowley G, Tanner R, Davoudi K, Shear B, Xu M, Karan K, O’reilly A, Ahmed S, Iskrenova Kirilova T, Ezekiel A, Shapland M, Mitra M, Krum T, Higginbotham G, Winayak R, Mustow J, Yorke L, Gibb C, Ilyas A, Pound M (Southmead Hospital, Bristol); Abouharb A*, Burke D, Khan A, Aimar K, Bennett J, Kerman-Fiore E, Rao S, Dayal A, Chawla S, Wadey L, Smith C, Desor K, Surti R, Gul Z, Gourgiotis V, Kaselampao LM, Nguik C, Agrawal R, Liaw QY, Mckeown E, Hassan A, Krishnan K, Glover HO, Devassy M, Ogunyanwo D, Dickenson G, Elder G, Valdez G, White B, Hamilton I, Shah S, Sinha S, Punwani K, Peat I, Hayat M, Khalid N, Frankel E, Macgowan H, Braka A, Byfield A, Brooks J, Kashif A, Archer R, Akkaya S, Sood M, Akther S, Birkett O, Kadri T, Abdulmula S, Psefteli P (St James University Hospital, Leeds); Chadha S*, Reese G, Mark C, Asunramu M, Hess T, Mehta S, Tsang C, Syed K, Alocious A, Steinruecke M (St Mary's Hospital IOW); Ashley M*, Newton R, Wainwright J, Ayathamattam J, Wong H (St Richards Hospital (Chichester)); Elahi Z*, Fleming M, Ali T, Lloyd D, Brooks K, Kwon C, Mckerrell A, Jamadar T, Barcelona MD, Polluk D, Kwon J, Henry F, Ayirookuzhi S, Mwipatayi M, Cheruiyot M, Adam S, Cruickshank T, Gollub K, Revell M, Taiwo S, Atchade A, Harewood A (Stoke Mandeville Hospital); Niina A*, Bosanquet D, Mckeon-Williams O, Szakmany T, Badhams E, Christensen N, Sammut J (THE GRANGE UNIVERSITY HOSPITAL); Tay N*, Pollok JM, Raptis D, Varcada M, Ganesananthan S, Chandrasekar R, Deshpande A, Ghoora LP, Hogg G, Staubli S, Banerjee M, Sunil Menon S, Sanz Pena A, Rana R, Mohamed A, Crabtree P, Flower A, Banerjee A, Talabi Y, Bishop N, Kupfuwa N, Ilangovan D, Qureshi H (The Royal Free Hospital); Minty I*, Baron R, Greasley L, Birkenhead P, Baron R, Shahid M, Rajasivam N, Chu WL, Punnoose N, Sundaresan V, El-Galley M, Prasansapakit K, Mitra S, Garlick M, Prakash M (The Royal Liverpool University Hospital); Gao H*, Parmar C, Lee F, Aich A, Ismail A, Kirupaharan M, Khan S, Patel K, Mclaverty D, Hammerton G, Walford H, Roberts H, Cautivo P (The Whittington Hospital); Abdel Kader H*, Kimble A, Manaf K, Mackonochie R, Mitchell V, Ghaznavi S, Taheem A, Firth E, Chandler H, Eldessouki K, Fyfe J, Bhat M, Cavlin J, Pearce E, Patterson S, Johnson C, El-Badawi K, Gariballa M, Hale E, Younis H, Brackenbury E, Chappell A, Poulton T, Dawson E (Torbay Hospital); Murray A*, Mcallister I, Hassan A, Cunningham B, Chan-A-Sue T, Rajpal N, Keane P, Walsh K, Mcgeoghan L, Neeson M, Brown H, Adams SM, Ladha S, Walsh A (Ulster Hospital); Alradhawi M*, Tarighi R, Miah MI, Dawas K, Mojadady A, Priestman-Degano A, Vellore Sasikumar SK, Suresh B, Fornasiero M, Csvila D, Kumar A, Adil ST, Adil HS, Kataria U, Jaibaji R, Patel N, Goch A, Quaye P, Gupte A, Mustafa H, Otti V (University College Hospital); Dewantoro D*, Ali M, Sood M, Wright H (University Hospital Ayr); Abusheba L*, Wong L, Docksey M, Abdinasir A, Karim MO, Kolaityte V, Vasileiadou Pelling M, Abdel Basit A, Pearson E, Hughes E, Millington AS (University Hospital Coventry and Warwickshire); Taylor L*, Borg C, Jagic K, Waheed A, Singh K, James V, Chowdhury T, Saxena A, Georgiou A (University Hospital Lewisham); Jones T*, Carpenter C, Hughes I, Aloul Z, Hanna-Davies C, Jacob A, Puthiyakunnel Saji A, Qureshi A, Ulfin Z, Dunlop M, Woods R, Hill N, Mohamed N, Vora A, Asharaf H, Maye E, Arif U, Srinivasan S, Prasad S, John B, Shivanand V, Singh A, Zahid A, Stewart E, Raketla M, Kokoricha O, Karwa D, Crisp J, Shehadeh R, Lee J, Webb B, Lepping S, Yusuf F, Pak E, Soo B, Pemmasani P, Goyal S (University Hospital of Wales); Binti Azad Bashir AB*, Komolafe S, Naeem A, Anbananthan HR, Au Yong LK (University Hospital Wishaw); Mookerjee S*, Ward N, Aniq M, Davis J, Al-Sukaini A, Anthony J (West Suffolk Hospital); Boyle J*, Laird A, Speake D, Beagan C, Stewart S, El-Koubani O, Chan R, Viswanath M, Janssens S, Shah I, Nguyen V, Mckee E, Ng CW, Balać M, Suresh Kumar DI, Seeva Balan S, Kirk R, Miles B, Kovacic LA, Collins VG, Low J, Sim W, Chua SJ, Narayanan N, Lee TY, Lo CMN (Western General Hospital, Edinburgh); Chauhan M*, Elkafsi J, Rasul S, Yammine R, Sattar Z, Dixit D (Wexham Park hospital); Rahman N*, Davies J, Dindyal S, Osborne L, Nip L, Wilding de Miranda N, Stevens A, Al-Diri B, Lim A, Kallikas G, Kim Y, Anjum M, Jeyapahan P, Hakim M, Singh S, Jamileh E, Sohrabi C, Baek SH, Sadik N, Mohammed A (Whipps Cross Hospital); Aje D*, Clifford R, O'halloran C, Mahajan K, Darke N (Whiston Hospital); Lloyd S*, Mlotshwa M, Melhuish K, Derex-Briggs J, Bell M, Varma P, Fray K, Garg Y (Worthing); Datta K, Mantel-Cooper T, Goodfellow M, Kazi B (Wrexham Maelor Hospital); Sievers N*, Telford K, Almansoor ZR, Craddock K, Tan JL, St John L, Singhania A, Dosani S, Mughal S, Bokhari N, Brooks L (Wythenshawe Hospital); Laid I*, Lala A, Ong CS, Wakefield S, Phillips S, Unwin H (Ysbyty Gwynedd, Bangor).

**References**

1. Collaborative S. Students' participation in collaborative research should be recognised. *Int J Surg* 2017; **39**: 234-7.

2. Beattie WS, Lalu M, Bocock M, et al. Systematic review and consensus definitions for the Standardized Endpoints in Perioperative Medicine (StEP) initiative: cardiovascular outcomes. *Br J Anaesth* 2021; **126**(1): 56-66.

3. Harris PA, Taylor R, Minor BL, et al. The REDCap consortium: Building an international community of software platform partners. *J Biomed Inform* 2019; **95**: 103208.

4. Jakobsen JC, Gluud C, Wetterslev J, Winkel P. When and how should multiple imputation be used for handling missing data in randomised clinical trials - a practical guide with flowcharts. *BMC Med Res Methodol* 2017; **17**(1): 162.
